# Repertoire Analysis of Antibody CDR-H3 Loops Suggests Affinity Maturation Does Not Typically Result in Rigidification

**DOI:** 10.3389/fimmu.2018.00413

**Published:** 2018-03-02

**Authors:** Jeliazko R. Jeliazkov, Adnan Sljoka, Daisuke Kuroda, Nobuyuki Tsuchimura, Naoki Katoh, Kouhei Tsumoto, Jeffrey J. Gray

**Affiliations:** ^1^Program in Molecular Biophysics, Johns Hopkins University, Baltimore, MD, United States; ^2^Department of Informatics, School of Science and Technology, Kwansei Gakuin University, Sanda, Hyogo, Japan; ^3^Department of Bioengineering, School of Engineering, The University of Tokyo, Tokyo, Japan; ^4^Medical Device Development and Regulation Research Center, School of Engineering, The University of Tokyo, Tokyo, Japan; ^5^Laboratory of Medical Proteomics, The Institute of Medical Science, The University of Tokyo, Tokyo, Japan; ^6^Department of Chemical and Biomolecular Engineering, Johns Hopkins University, Baltimore, MD, United States; ^7^Institute for NanoBioTechnology, Johns Hopkins University, Baltimore, MD, United States; ^8^Sidney Kimmel Comprehensive Cancer Center, Johns Hopkins University, Baltimore, MD, United States

**Keywords:** antibody repertoires, affinity maturation, complementarity determining regions, conformational flexibility, rigidity theory, pebble game algorithm, RosettaAntibody, molecular dynamics simulations

## Abstract

Antibodies can rapidly evolve in specific response to antigens. Affinity maturation drives this evolution through cycles of mutation and selection leading to enhanced antibody specificity and affinity. Elucidating the biophysical mechanisms that underlie affinity maturation is fundamental to understanding B-cell immunity. An emergent hypothesis is that affinity maturation reduces the conformational flexibility of the antibody’s antigen-binding paratope to minimize entropic losses incurred upon binding. In recent years, computational and experimental approaches have tested this hypothesis on a small number of antibodies, often observing a decrease in the flexibility of the complementarity determining region (CDR) loops that typically comprise the paratope and in particular the CDR-H3 loop, which contributes a plurality of antigen contacts. However, there were a few exceptions and previous studies were limited to a small handful of cases. Here, we determined the structural flexibility of the CDR-H3 loop for thousands of recent homology models of the human peripheral blood cell antibody repertoire using rigidity theory. We found no clear delineation in the flexibility of naïve and antigen-experienced antibodies. To account for possible sources of error, we additionally analyzed hundreds of human and mouse antibodies in the Protein Data Bank through both rigidity theory and B-factor analysis. By both metrics, we observed only a slight decrease in the CDR-H3 loop flexibility when comparing affinity matured antibodies to naïve antibodies, and the decrease was not as drastic as previously reported. Further analysis, incorporating molecular dynamics simulations, revealed a spectrum of changes in flexibility. Our results suggest that rigidification may be just one of many biophysical mechanisms for increasing affinity.

## Introduction

Antibodies are proteins produced by the B cells of jawed vertebrates that play a central role in the adaptive immune system. They recognize a variety of pathogens and induce further immune response to protect the organism from external perturbation. Molecules that are bound by antibodies are referred to as antigen and are recognized by the antibody variable domain (Fv), which is comprised of a variable heavy (V_H_) and light (V_L_) domain. To overcome the challenge of recognizing a vast array of targets—the number of antigens being far greater than the number of antibody germline genes—antibodies rely on combinatoric and genetic mechanisms that increase sequence diversity (1–3). Starting from a limited array of germline genes, a naïve antibody is generated by productive pairing of a randomly recombined V_H_, assembled from V-, D-, and J-genes on the heavy locus, and randomly recombined V_L_, assembled from V- and J-genes on the kappa and lambda loci (1). Next, in a process known as affinity maturation, iterations of somatic hypermutation are followed by selection to evolve the antibody in specific response to a particular antigen. This evolution results in the gradual accumulation of mutations across the entire antibody, with higher mutation rates in the six complementarity determining regions (CDRs) than in the framework regions (FRs) (4, 5). The CDRs are hypervariable loops comprising a binding interface on the Fv domain beta-sandwich framework, with three loops contributed by each chain; the light chain CDRs are denoted as L1, L2, and L3 and the heavy chain CDRs are denoted as H1, H2, and H3. The five non-H3 CDRs can be readily classified into a discrete amount of canonical structures (6–10) because they possess limited diversity in both sequence and structure. The CDR-H3 on the other hand is the focal point of V(D)J recombination, resulting in exceptional diversity of both structure and sequence. While all CDRs contribute to antigen binding, the diverse CDR-H3 is often the most important CDR for antigen recognition (11–15). Thus, to understand the role of B cells in adaptive immunity and how they evolve antibodies capable of binding specific antigens, we must first understand the effects of affinity maturation on the CDRs, and in particular on the CDR-H3.

Over the past 20 years, the effects of affinity maturation have been studied with an assortment of experimental and computational methods. X-ray crystallography has been used to compare antigen-inexperienced (naïve) and antigen-experienced (mature) antibodies with both antigen present and absent. Analysis of the catalytic antibodies 48G7, AZ-28, 28B4, and 7G12 showed a 1.2 Å average increase in Cα root-mean-square deviation (RMSD) of the CDR-H3 upon antigen binding in the naïve over that of the mature antibody, whereas motion in the other CDRs varied (16–20). Beyond structural studies, surface plasmon resonance has been used to assess the energetics and association/dissociation rate constants of antibody–antigen binding. Manivel et al. studied a panel of 14 primary (naïve) and 11 secondary (mature) response anti-peptide antibodies, observing that affinity maturation resulted in increases in the association rate and corresponding changes in the entropy of binding (21). Schmidt et al. saw the opposite when studying a broadly neutralizing influenza virus antibody, observing that affinity maturation resulted primarily in a decrease in the dissociation rate, with little effect on the association rate (22). Isothermal calorimetry (ITC) has also been used to determine antigen-binding energetic, including the enthalpic and entropic contributions. For nine anti-fluorescein antibodies, including 4-4-20 and eight anti-MPTS antibodies, ITC results revealed diverse effects of affinity maturation: 14 of 17 mature antibodies bound antigen in an enthalpically favorable and entropically unfavorable manner, yet 3 of 17 showed the opposite, with entropically favorable and enthalpically unfavorable binding energetics (23, 24). Three-pulse photon echo peak shift (3PEPS) spectroscopy has been used to quantify dynamics of chromophore-bound antibodies on short timescales of femto- to nanoseconds. 3PEPS spectroscopy results from a panel of 18 antibodies showed that mature antibodies can possess a range of motions from small rearrangements such as side-chain motions to large rearrangements such as loop motions (23–25). In a specific comparison of naïve vs. mature, for the 4-4-20 antibody, the mature antibody was found to have smaller motions, i.e., to be more rigid, than naïve (23–28). Antibody dynamics have also been studied by hydrogen–deuterium exchange mass spectroscopy (HDX-MS), which in contrast to 3PEPS probes timescales of seconds to hours. Comparison of three naïve and mature anti-HIV antibodies showed changes in CDR-L2/H2, but not in CDR-H3 dynamics (29). Finally, molecular dynamics (MD) simulations have been used to study antibody dynamics on intermediate timescales of nano- to microseconds. MD simulations showed rigidification and reduction of CDR-H3 loop motion upon maturation for seven studied naïve/mature antibodies, with two exceptions, depending on the specific study (22, 28, 30–34). In an orthogonal protein design approach to examine the CDR-H3 loop flexibility, Babor et al. and Willis et al. found that naive antibody structures are more optimal for their sequences, when considering multiple CDR-H3 loop conformations (35, 36). In sum, past studies focusing on the effects of affinity maturation on CDRs have found evidence suggesting that mature antibodies have more structural rigidity and less conformational diversity than their naïve counterparts (16, 18, 19, 23–27).

With recent growth in the number of antibody structures deposited in the Protein Data Bank (PDB) and development of homology models from high-throughput sequencing of paired V_H_–V_L_ genes in B cells, we now have the datasets necessary to test the rigidity hypothesis on a large scale. Prior studies, usually focused on a few antibodies at time, generally support the hypothesis that affinity maturation rigidifies the CDR-H3 loop. Thus, we hypothesize that this effect should be observable in a repertoire-scale study of thousands of antibodies. We first analyzed thousands of recently determined RosettaAntibody homology models of the most common antibody sequences found in the human peripheral blood cell repertoire (37). We estimated the structural flexibility of the CDR-H3 loop by applying graph theoretical techniques based on mathematical rigidity theory, namely the Floppy Inclusions and Rigid Substructure Topography (FIRST) and extensions of the Pebble Game (PG) algorithms to determine backbone degrees of freedom (DOFs). Surprisingly, we found no difference in the CDR-H3 loop flexibility of the naïve and mature antibody repertoires. We considered alternative explanations for our results, which were incongruent with past studies, by expanding our analysis to a large set of antibody crystal structures, including several previously characterized antibodies, and extending our methods to include other measures of flexibility, such as B-factors and MD simulations. By all analysis methods, we found mixed results: some antibodies’ CDR-H3 loops were more flexible after affinity maturation whereas others’ became less flexible. In summary, we find that while affinity maturation can modulate antibody binding activity by reducing CDR-H3 structural flexibility, it does not necessarily do so.

## Materials and Methods

### Immunomic Repertoire Modeling

Briefly, RosettaAntibody is an antibody modeling approach that assembles homologous structural regions into a rough model and then refines the model through gradient-based energy minimization, side-chain repacking, rigid-body docking, and *de novo* loop modeling of the CDR-H3. The approach is fully detailed in Ref. (38, 39). In a typical simulation, ~1,000 models are generated and the 10 lowest-energy models are retained. The immunomic repertoire we analyzed is from DeKosky et al. (37). In that study, models were generated for each of the ~1,000 most frequently occurring naïve and mature antibody sequences from two donors (a total of ~20,000 models representing the ~2,000 most frequent antibodies).

### Structural Rigidity Determination

The flexibility or rigidity of the CDR-H3 loop backbone was determined by using several extensions of the PG algorithm (40–43), initially developed in Ref. (40), and method FIRST (44); we refer to here as FIRST-PG. This approach can determine flexible and rigid regions in a protein and quantify the internal conformational DOFs from a single protein conformational snapshot. FIRST generates a molecular constraint network (i.e., a graph) consisting of vertices (nodes) representing atoms and edges (interactions representing covalent bonds, hydrogen bonds, hydrophobic interactions, etc.). Each potential hydrogen bond is assigned with energy in kcal/mol which is dependent on donor-hydrogen acceptor geometry. FIRST is run with a selected hydrogen-bonding energy cutoff, where all bonds weaker than this cutoff are ignored in the network. On the resulting network, the well-developed mathematical and structural engineering concepts (45) of flexibility and rigidity of molecular frameworks and the PG algorithm are then used to identify rigid clusters, flexible regions, and overall available conformational DOFs. For a given antibody structure, DOFs for the protein backbone of the CDR-H3 loop were calculated at every hydrogen-bonding energy cutoff value between 0 and −7 kcal/mol in increment steps of 0.01 kcal/mol. This calculation was repeated for every member of that antibody ensemble (i.e., 10 lowest-energy models of the ensemble) and finally, at each energy cutoff, the DOF count was averaged over the entire ensemble.

For a given energy cutoff and a given member of the ensemble, the DOF count for the CDR-H3 loop (residues 95–102) was obtained using a special PG operation which calculates the maximum number of pebbles that can be gathered on the backbone atoms (Cα, C, N) of the CDR-H3 loop (40). The PG algorithm starts with the constrained molecular graph and generates a directed multigraph, where available free pebbles are absorbed one by one by independent edges (constraints). Each pebble represents one of six DOF associated with an atom. After PG completion, the remaining free pebbles can be collected on the CDR-H3 backbone (i.e., a subgraph in the constrained network) represent its conformational DOF count.

### DOF Scaling

To compare flexibility across CDR-H3 loops of different lengths, the DOF metric computed above is scaled by a theoretical maximum DOF. We define $s\text{DOF}=\frac{\text{DOF}}{2L+6}$, where, 2*L* (the loop length in residues) represents the backbone DOFs (torsion angles: ϕ, ψ), and 6 represents the trivial, but ever-present rigid-body DOFs (i.e., combination of rotations and translations in 3D).

### Area under Curve (AUC) Calculation

The AUC is approximated by simple numerical integral (akin to trapezoidal integration), where the first term defines a rectangle and the second term defines a triangle:

$$\text{AUC}\equiv \sum (x_{i}-x_{i-1})\cdot y_{i-1}+\frac{1}{2}(x_{i}-x_{i-1})(y_{i}-y_{i-1}).$$

### Crystallographic Dataset

On June 27th, 2017, a summary file was generated from the Structural Antibody Database (SAbDab) (46), using the “non-redundant search” option to search for antibodies with maximum 99% sequence identity, paired heavy and light chains, and a resolution cutoff of 3.0 Å. The summary file, containing 1,021 antibodies, was used as input to a SAbDab download script which yielded corresponding sequences, Chothia-numbered PDBs, and IMGT data (on occasion this had to be updated to match the reported germline in the IMGT 3Dstructure-DB) (47). The structures were further pruned: structures were omitted if there were unresolved CDR-H3 residues, as this would preclude flexibility calculations, or if the antibody was neither human nor mouse, as this would prevent alignment to germline. Prior to analysis, structures were truncated to the Fv region (removing all residues, but light chain residues numbered 1–108 and heavy chain residues numbered 1–112, in Chothia numbering) and duplicate and non-antibody (for example, bound antigen) chains were removed. A total of 922 antibody crystal structures were analyzed. The following CDR definitions were used throughout this paper, in conjunction with the Chothia numbering scheme: L1 spans light chain residue numbers 24–34, L2 spans 50–56, L3 spans 89–97, H1 spans heavy chain residue numbers 26–35, H2 spans 50–56, and H3 spans 95–102.

### Alignment to Germline

The germline of each antibody was determined by IMGT lookup (47). Then, BLASTP (version 2.2.29+) with the BLOSUM50 scoring matrix was used to align the antibody variable region heavy and light sequences to corresponding germline sequences (IGHV, IGKV, and IGLV loci only, downloaded from IMGT). The number of mismatches according to BLAST was considered as the number of amino acid mutations from germline. Table S1 in Supplementary Material details the PDB ID, CDR-H3 length, number of heavy chain mutations, number of light chain mutations, heavy germline gene, and light germline gene data for each structure in the dataset.

### B-Factor *z*-Score Calculation

Temperature factors (B-factors) were extracted for all Cα atoms in the variable region of the antibody heavy chain (V_H_, Chothia numbering 1–112). The arithmetic mean and sample SD values were calculated for the B-factors. For each Cα atom in the CDR-H3 region, residue numbers spanning 95–102 under the Chothia numbering convention (11), the *z*-score was calculated as $\frac{\left(x-\mu \right)}{\sigma }$, where *x* is the B-factor of the current Cα atom and μ and σ are the mean and SD of B-factors for all Cα atoms in the V_H_, respectively. PDB IDs 2NR6 and 3HAE were excluded from B-factor analysis because all reported B-factors were identical and so the *z*-scores were 0 by definition.

### B-Factor *z*-Score Distribution Randomization Testing

To test whether two observed B-factor distributions arose from the same underlying distribution, we turned to randomization testing. First, we computed the difference of the observed distribution means. Next, we pooled the data from the two distributions (e.g., CDR-H3 loop B-factor *z*-scores) and randomly sampled the pooled data to create two simulated distributions (e.g., randomly assigning *z*-scores to either the naïve or mature category). Finally, we computed the simulated difference of the randomized distribution means. This process was repeated 10,000 times, so we could identify the fraction of random distributions with differences greater than the observed. Since, this process is stochastic and does not exhaustively sample all permutations of the data, it was further repeated 10 times to acquire a SD.

### Rosetta Relaxation and Ensemble Generation

Antibody structural ensembles with 10 members were generated using either the Rosetta FastRelax (48, 49) or Rosetta KIC protocol (50), and Rosetta version 2017.26-dev59567 was used for all simulations (corresponding to weekly release version 2017.26). The Rosetta FastRelax protocol consists of five cycles of side-chain repacking and gradient-based energy minimization in the REF2015 version of the Rosetta energy function (51). Thus, FastRelax ensembles explore the local energy minimum of the crystal structure. KIC ensembles are more diverse and representative of RosettaAntibody homology models: each ensemble member was generated by running the CDR-H3 refinement step of the RosettaAntibody protocol, consisting of V_H_–V_L_ docking, CDR-H3 loop remodeling, and all-CDR loop minimization (38, 39). Sample command lines are given in Supplementary Material. The structural ensembles produced by both FastRelax and KIC were used for rigidity analysis. For technical reasons, 6 targets could not be analyzed from the FastRelax ensemble, and 177 targets from the KIC ensemble were omitted due to non-trivial incompatibilities between the input structure numbering and Rosetta’s internal antibody numbering scheme and a computing cluster time limitation. The excluded targets were randomly distributed and likely would not affect the conclusions.

### MD Simulations

The Fv regions were retrieved from the original PDB files. The MD simulations were performed using the NAMD 2.12 package (52) with the CHARMM36m force field and the CMAP backbone energy correction (53). The truncated Fv structures were solvated with TIP3P water in a rectangular box such that the minimum distance to the edge of the box was 12 Å under periodic boundary conditions. Na or Cl ions were added to neutralize the protein charge, then further ions were added corresponding to a salt solution of concentration 0.14 M. The time step was set to two Fs throughout the simulations. A cutoff distance of 10 Å for Coulomb and van der Waals interactions was used. Long-range electrostatics was evaluated through the Particle Mesh Ewald method (54).

The initial structures were energy minimized by the conjugate gradient method (10,000 steps), and heated from 50 to 300 K during 100 ps, and the simulations were continued by 1 ns with NVT ensemble, where protein atoms were initially held fixed whereas non-protein atoms freely moved, gradually releasing the whole system to facilitate a stable simulation over the 1 ns. Further simulations were performed with NPT ensemble at 300 K for 200 ns without any restraints other than the SHAKE algorithm to constrain bonds involving hydrogen atoms. The last 180 ns of each trajectory were used for the subsequent clustering analyses. Similar to a previous work (55), a total of 2,000 evenly spaced frames from each trajectory were clustered based on RMSD of the Cα and Cβ atoms using the K-means clustering algorithm implemented in the KCLUST module in the MMTSB tool set (56). The cluster radius was adjusted to maintain 20 clusters in each trajectory. The structure closest to the center of each cluster was chosen as a representative structure of each cluster. The 10 representative structures were chosen from the top 10 largest clusters and these representative structures were energy minimized by the conjugate gradient method (10,000 steps) in a rectangular water box. The minimized antibody Fv structures were used as the inputs for the rigidity analysis.

Root-mean-square quantities of the MD trajectories were calculated based on the past 180 ns trajectories. After superposing Cα atoms of the FR of the heavy chain (FR_H_) of each snapshot onto Cα atoms of FR_H_ of the reference structures (i.e., crystal structures), Cα-RMSD of the CDR-H3 loop was calculated as the time average. Similarly, after superposing Cα atoms of entire Fv domains of each snapshot onto those of the reference structures, the root-mean-square fluctuation (RMSF) of a residue *i* was defined as the time average:

$${\text{RMSF}}_{i}=\sqrt{\langle {\left(x_{i}-\langle x_{i}\rangle \right)}^{2}\rangle }$$

where *x_i_* is the distance between the Cα atom of the snapshots at a given time and the Cα atom of the *i*th residue of the reference structures (57).

## Results

### Immunomic Repertoire Reveals No Difference in Flexibility between Naïve and Mature CDR-H3 Loops

We initially asked whether CDR-H3 loop rigidification, having been observed in many past studies, was present in a large set of antibodies derived from human peripheral blood cells. Previously, DeKosky et al. used RosettaAntibody to model the structures of 1,911 common antibodies found in the peripheral blood cells of two human donors (37). Paired V_H_–V_L_ sequences were derived from either CD3^−^CD19^+^CD20^+^CD27^−^ naïve B cells or CD3^−^CD19^+^CD20^+^CD27^+^ antigen-experienced B cells (mature) isolated from peripheral mononuclear cells. RosettaAntibody structural models were created by identifying homologous templates for the CDRs, V_H_–V_L_ orientation, and FRs; assembling the templates into one model; *de novo* modeling the CDR-H3 loop; rigid-body docking the V_H_–V_L_ interface; side-chain packing; and minimizing in the Rosetta energy function (38). Since *de novo* modeling of long loops is challenging, DeKosky et al. limited their antibody set to the more tractable subset of antibodies with CDR-H3 loop lengths under 16 residues. They compared their models for seven human germline antibodies with solved crystal structures and found models had under 1.4 Å backbone RMSD for the FR and under 2.4 Å backbone RMSD for the CDR-H3 loop.

**Figure 1:**
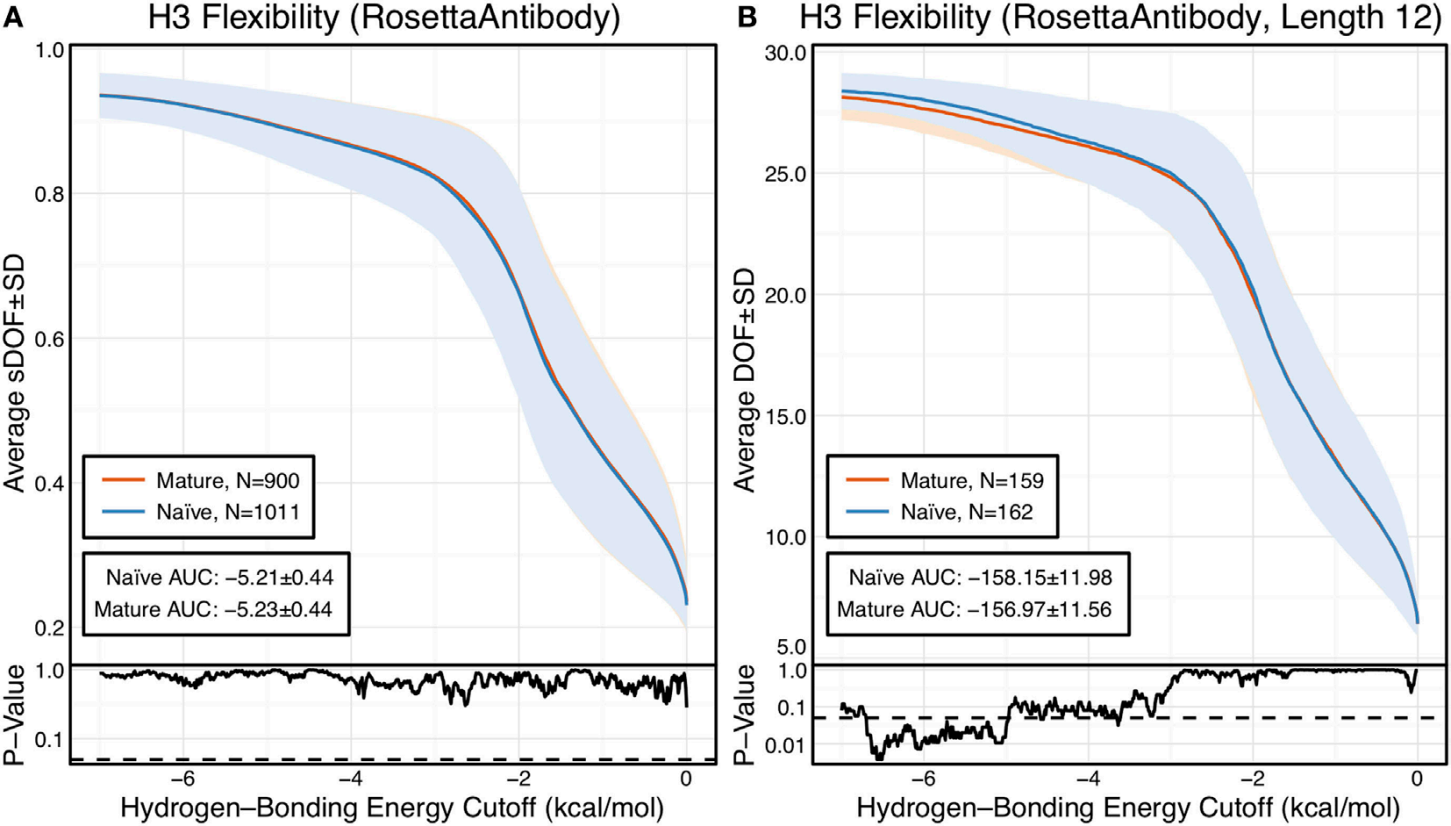
CDR-H3 loop flexibility analysis of the immunomic antibody set reveals that no difference in naïve (blue) and mature (red) antibodies. Floppy inclusions and rigid substructure topography-Pebble Game was used to determine the degrees of freedoms (DOFs) as a function of hydrogen-bonding energy cutoff in RosettaAntibody models of the 1,911 most frequent public antibodies. Results were split, depending on whether the antibody was naïve or mature, as determined by B-cell surface receptors, and the mean DOFs were calculated along with the SD, shown in a lighter shade of the respective color. Subplots, below each main plot, show the *p*-value computed by a two-sample Kolmogorov–Smirnov (KS) test comparison of the naïve and mature DOFs distributions for each hydrogen-bonding energy cutoff, with null hypothesis being that the distributions are the same. A dashed line indicates a *p*-value of 0.05. **(A)** To permit comparison across loops of multiple lengths, the DOFs were scaled to a theoretical maximum for each length (a value of 1 indicates all DOFs are available, whereas a value of 0 indicates no DOFs are available). We found the scaled DOFs to be similar for both naïve and mature antibodies, quantified by the KS test *p*-values and area under the curve (AUC) ± SD: −5.21 ± 0.44 and −5.23 ± 0.44, respectively. **(B)** To exclude length effects on flexibility calculations, we compared DOFs for the most popular length (12 residues). We found the naïve AUC ± SD at −158.15 ± 11.98 and mature AUC ± SD at −156.97 ± 11.56 to be similar. The distributions appear similar at cutoffs between 0 and −5.0 kcal/mol, according to the KS test *p*-values.

We used the FIRST-PG method (40, 44) to estimate flexibility from the RosettaAntibody homology models, determining the number of backbone DOFs for the CDR-H3 loop as each hydrogen bond is broken in order from weakest to strongest. FIRST models the antibody as a molecular graph where nodes represent atoms and edges represent atomic interactions. An extension of the PG algorithm uses this molecular graph to compute the DOFs of the CDR-H3 loop. To mitigate the effects of homology modeling, inaccuracies on the FIRST-PG analysis, we used an ensemble of 10 lowest-energy RosettaAntibody models. FIRST-PG analysis on structural ensembles has been shown to predict hydrogen–deuterium exchange and protein flexibility (51). To account for varying CDR-H3 loop lengths, we scaled the calculated DOFs by a theoretical maximum value (see Materials and Methods). Figure 1A shows a curve of the scaled DOFs averaged over all naïve or mature antibodies as a function of the hydrogen-bonding energy cutoff used in the FIRST-PG analysis. At a cutoff of 0 kcal/mol, all hydrogen bonds are intact and the average CDR-H3 loop-scaled DOFs are about 20% of the theoretical maximum. Moving from right to left on the plot increases the minimum energy cutoff for including interactions in the FIRST graph; effectively hydrogen bonds of increasing strength are “broken” and the available DOFs rise from 20 to above 90% of the maximum theoretical flexibility, while the loop becomes unstructured (unfolded) in FIRST.

We compared the DOFs distributions for naïve and mature antibodies at every hydrogen-bonding energy cutoff by two-sample Kolmogorov–Smirnov (KS) testing, with null hypothesis being that the two distributions are identical (Figure 1A). There is no difference in the average, scaled DOFs. To further quantify this comparison, we computed the average AUC plus-or-minus one SD for both antibody sets. The average AUC values are identical for the naïve (−5.21 ± 0.44) and mature antibody repertoires (−5.23 ± 0.44). This lack of difference persists (AUC − 158.15 ± 11.98 [naïve] vs. −156.97 ± 11.56 [mature]) when accounting for CDR-H3 loop length, by comparing loops of only length 12, the most popular length (Figure 1B), and so the observed similarity of DOFs in naïve and mature antibodies is not due to averaging over loops of different lengths. Thus, on the immunomic repertoire scale, we do not observe the difference in flexibility between naïve and mature antibodies predicted by the paratope rigidification hypothesis.

Before amending the rigidification hypothesis in light of these results, we considered several alternative explanations for our observations. First, we addressed whether the use of homology models for flexibility analysis introduced inaccuracies by analyzing a large set of antibody crystal structures and Rosetta-generated models from that set with varying quality, ranging from models with sub-angstrom backbone RSMD to models that may be several angstroms off (and more representative of an average homology model). Next, we addressed whether backbone DOFs, as calculated by FIRST-PG, were a good measure of flexibility, by assessing flexibility through two alternative measures: B-factors and MD simulations. Additionally, we addressed whether averaging flexibilities and comparing across many germlines affected results, by detailed flexibility analysis of previously studied naïve–mature antibody pairs and RosettaAntibody-modeled pairs.

### Only Small Flexibility Differences Are Observed between Naïve and Mature Antibodies in the Crystallographic Set

#### Preparation of an Antibody Crystal Structure Dataset

Of course, the strongest critique of the immunomic antibody set is that these models are only approximating the actual antibody structure. Thus, we applied FIRST-PG analysis to a large set of antibody crystal structures. We curated the set of all non-redundant mouse and human antibody crystal structures from SAbDab (46). To be consistent with the models produced by RosettaAntibody, we truncated the structure of each antibody to only the Fv domain, excluding other antibody regions or antigen. Then, we used IMGT/3Dstructure-DB (58) to identify the variable domain genes and determined the number of somatic mutations by aligning the sequence derived from the crystal structure to the IMGT-determined V-gene. We defined mature antibodies as those possessing at least one somatic mutation in either V-gene. Our full dataset has 922 antibodies of which 23 are naïve. CDR-H3 loop lengths and germline assignments are summarized in Table S1 in Supplementary Material. Summary statistics are plotted in Figures S1–S3 in Supplementary Material.

#### FIRST-PG Analysis of Crystal Structures

From the crystal structures, we created two sets of structural ensembles and assessed flexibility by FIRST-PG. Flexibility analysis has previously been shown to be more accurate on ensembles in comparison to analysis using single (snapshot) conformers (41, 59). Ensembles of 10 representative structures were generated from the initial crystal structure by using either Rosetta FastRelax (48) or refinement step of RosettaAntibody (38, 39), which we term KIC ensembles after the loop modeling algorithm used in refinement (50). Rosetta FastRelax samples structures around the crystallographic, local energy minimum, with typically <1 Å backbone RMSD, whereas the refinement step of RosettaAntibody samples a more diverse set of low-energy CDR-H3 loop conformations and V_H_–V_L_ orientations. Thus, FastRelax ensembles are representative of the crystal structures, whereas KIC ensembles are representative of RosettaAntibody homology models. By comparative FIRST-PG analysis of the two sets, we can assess the effects of modeling inaccuracies on flexibility analysis.

**Figure 2:**
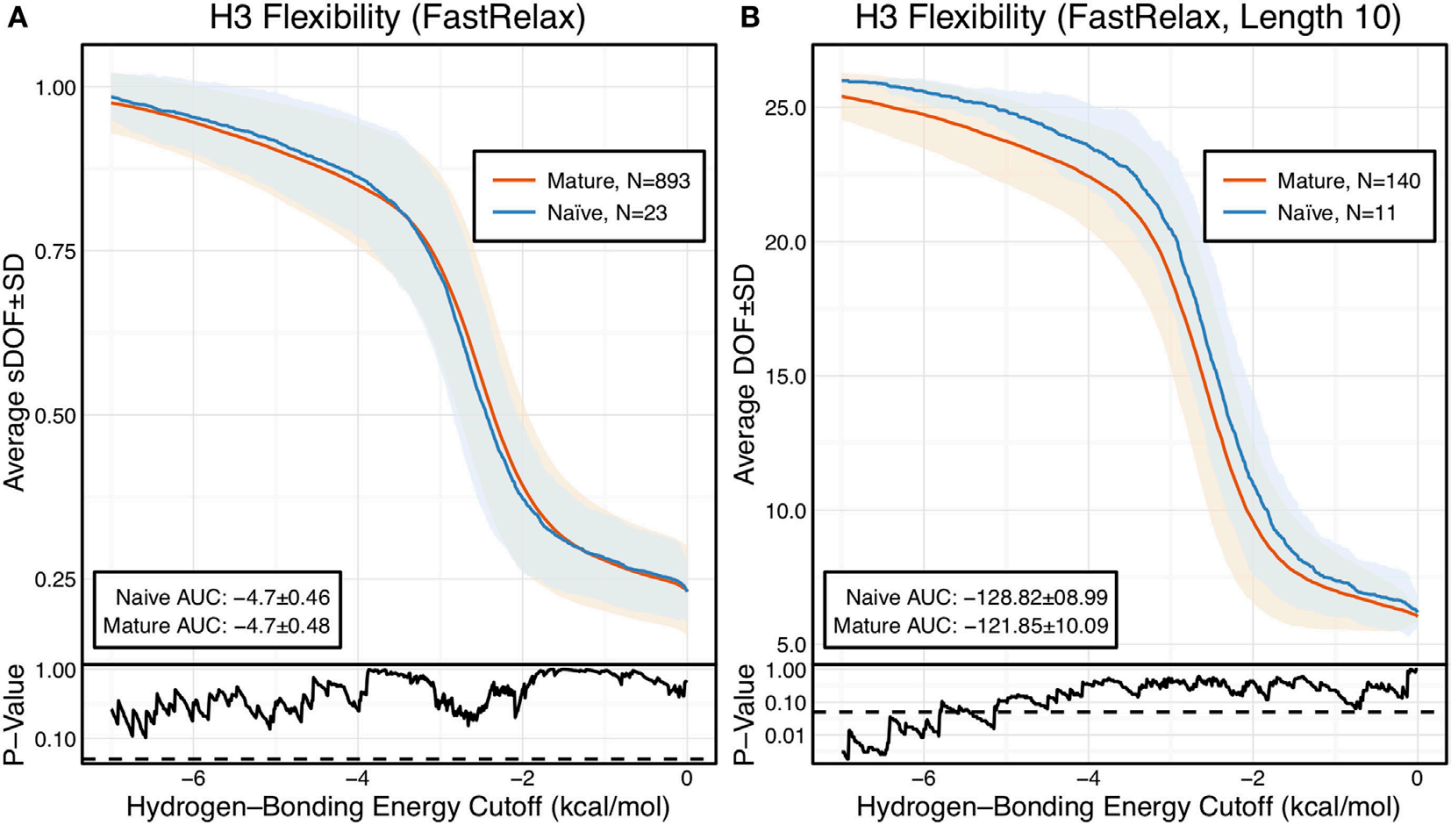
When accounting for length, CDR-H3 loop flexibility analysis of the crystallographic antibody set reveals naïve (blue) antibodies to be slightly more flexible than mature (red). Floppy Inclusions and Rigid Substructure Topography-Pebble Game was used to determine the degrees of freedoms (DOFs) as a function of hydrogen-bonding energy cutoffs in crystal structure ensembles created by Rosetta FastRelax. Results were split, depending on whether the antibody was naïve or mature, as determined by BLAST alignment to its germline V-genes, and the mean DOFs were calculated along with the SD, shown in a lighter shade of the respective color. Subplots, below each main plot, show the *p*-value computed by a Kolmogorov–Smirnov (KS) test comparison of the naïve and mature DOF distributions for each hydrogen-bonding energy cutoff, with null hypothesis being that the distributions are the same. A dashed line indicates a *p*-value of 0.05. **(A)** To permit comparison across loops of multiple lengths, the DOFs were scaled to a theoretical maximum for each length (a value of 1 indicates all DOFs are available whereas a value of 0 indicates not DOFs are available). We found the scaled DOFs to be similar for both naïve and mature antibodies, quantified by KS test *p*-values and the areas under the curve (AUCs) ± SD: −4.70 ± 0.46 and −4.70 ± 0.48, respectively. **(B)** To exclude length effects on flexibility calculations, we compared DOFs for the most popular length (10 residues). We found the naïve AUC ± SD at −128.82 ± 8.99 was greater than the mature AUC ± SD at −121.85 ± 10.09, but still within a SD. The distributions appear similar at cutoffs between 0 and −6.0 kcal/mol, according to the KS test *p*-values.

The scaled DOFs as calculated by FIRST-PG for FastRelax ensembles of antibody crystal structures are shown in Figure 2A. There are only minor differences between the naïve and mature flexibility curves, two-sample KS testing reveals insignificant *p*-values (?0.05) for all hydrogen-bonding energy cutoffs, and the AUC is similar for both sets (−4.70 ± 0.46 [naïve] vs. −4.70 ± 0.48 [mature]). Again, we considered the possibility that different distributions of loop lengths in the two sets obscures the affinity maturation contributions to flexibility. Therefore, we analyzed loops of length 10 (Figure 2B), the single most common length in the crystallographic set. When loops of a single length were compared, there was a separation between the naïve and mature sets, with the naïve antibody set average DOFs being consistently greater than the mature set, but not significantly so, except for some energy cutoffs below −5 kcal/mol, according to KS testing. As expected, the AUC values differ, but are within a SD (−128.2 ± 9.0 [naïve] vs. −121.9 ± 10.1 [mature]). We repeated FIRST-PG analysis for KIC ensembles of antibody crystal structures and observed similar results (Figure S4 in Supplementary Material): for scaled DOFs, the AUC was −5.91 ± 0.20 (naïve) vs. −5.81 ± 0.26 (mature) and, for loops of length 10 only, the AUC was −154.10 ± 4.80 (naïve) vs. −150.44 ± 7.73 (mature). Thus, there does not appear to be a large, consistent CDR-H3 loop flexibility difference across all antibody crystal structures analyzed.

#### B-Factor Analysis of Crystal Structures

However, we have not accounted for the possibility that backbone DOFs as calculated by FIRST-PG may not capture the effects of affinity maturation on CDR-H3 loop flexibility. Thus, we assessed loop flexibility as determined by atomic temperature factors or B-factors. In protein crystal structures, B-factors measure the heterogeneity of atoms in the crystal lattice. Thus, rigid regions have lower B-factors as they are more homogenous throughout the crystal, whereas flexible regions have higher B-factors as they are less homogenous throughout the crystal. B-factors are also affected by crystal resolution, so we cannot compare raw values across structures of varying resolution. Instead, we computed a normalized B-factor *z*-score, which has 0 mean and unit SD for each antibody chain. Finally, to account for different CDR-H3 loop lengths, we averaged the B-factor *z*-scores for the CDR-H3 loop residues.

**Figure 3:**
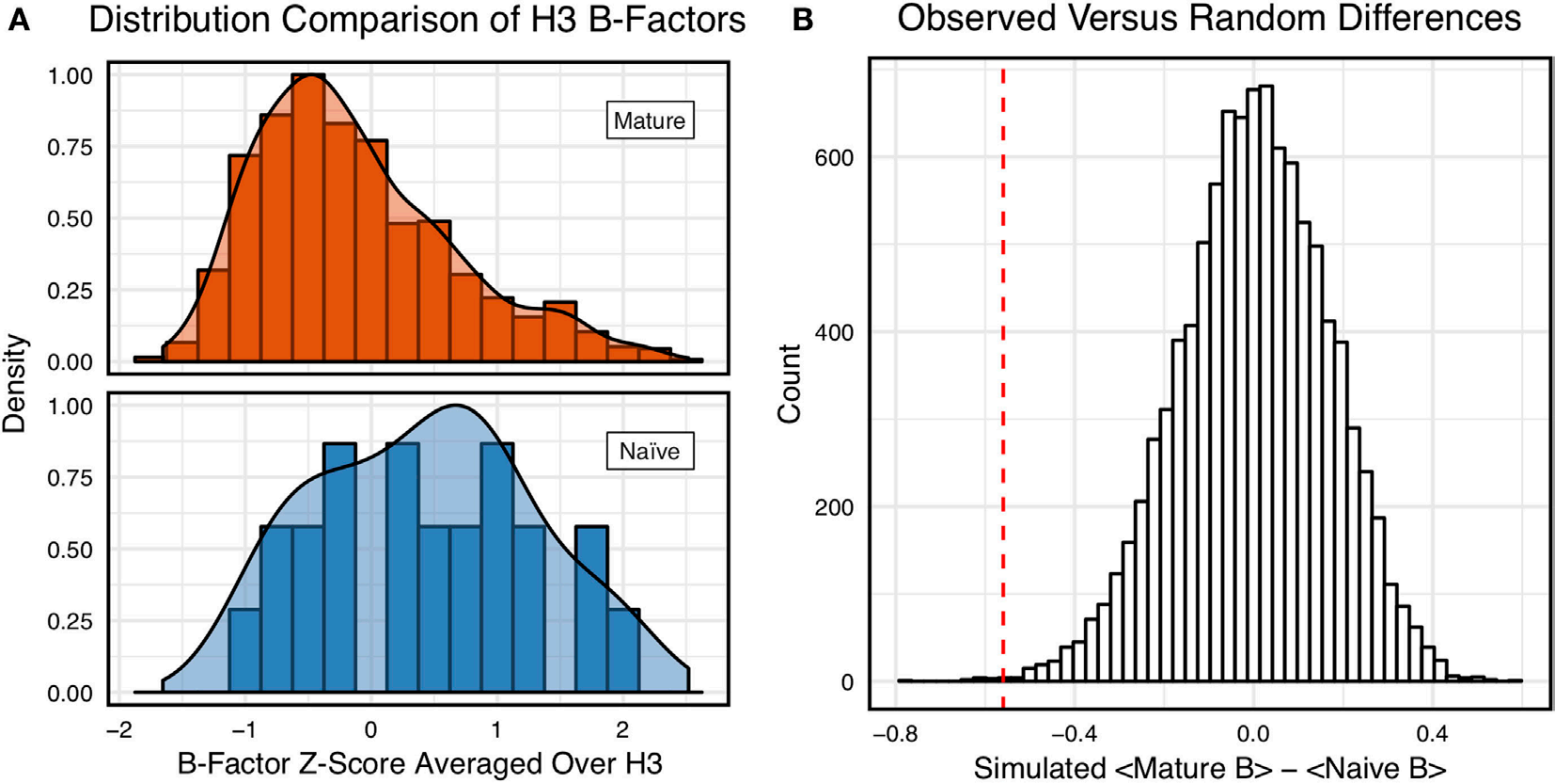
Comparison of the distribution of average CDR-H3 loop B-factor *z*-scores in antibody crystal structures suggests that naïve are more flexible than mature. **(A)** Distributions of average CDR-H3 loop B-factors for the crystallographic set of antibodies are distinct for the mature (orange) and naïve (blue) sets. The mature antibody CDR-H3 loops have lower B-factors than the naïve, corresponding to more rigidity. Bars show binned counts in intervals of 0.25. Both the bars and smoothed densities are normalized so the maximum value is 1. A two-sample Kolmogorov–Smirnov test confirms different underlying distributions with a *p*-value of 0.006 and maximum vertical deviation, *D*, of 0.36. **(B)** The observed difference in distribution means is difficult to replicate by random chance, occurring only 6.6 ± 2.6 times out of 10,000 simulations. Comparing the observed difference in means (red line, dashed) to simulated differences (white bars) acquired by randomly assigning B-factor values from the original distributions to either a naïve or mature set, in the observed numbers (*N*_mature_ = 897 and *N*_naive_ = 23), before computing the difference in means.

Figure 3A shows the distributions of B-factor *z*-scores averaged over the CDR-H3 loop residues of naïve and mature antibodies. Both distributions span a similar range and overlap significantly, with the naïve curve peak shifted toward higher values than the mature. The majority of the naïve CDR-H3 loop B-factor *z*-score averages were positive (65%), whereas the majority of the mature CDR-H3 loop B-factor *z*-score averages were negative (64%). To address the question whether these distributions arose from the same underlying distribution we turned to randomization testing, as described in Section “Materials and Methods.” The observed difference in distribution means is matched by only 0.066 ± 0.026% of simulated differences (Figure 3B), indicating that naïve and mature distributions are likely distinct. Furthermore, a two-sample KS test confirms the distributions to be distinct, with a maximum vertical deviation, *D*, of 0.36 and a *p*-value of 0.006.

**Figure 4:**
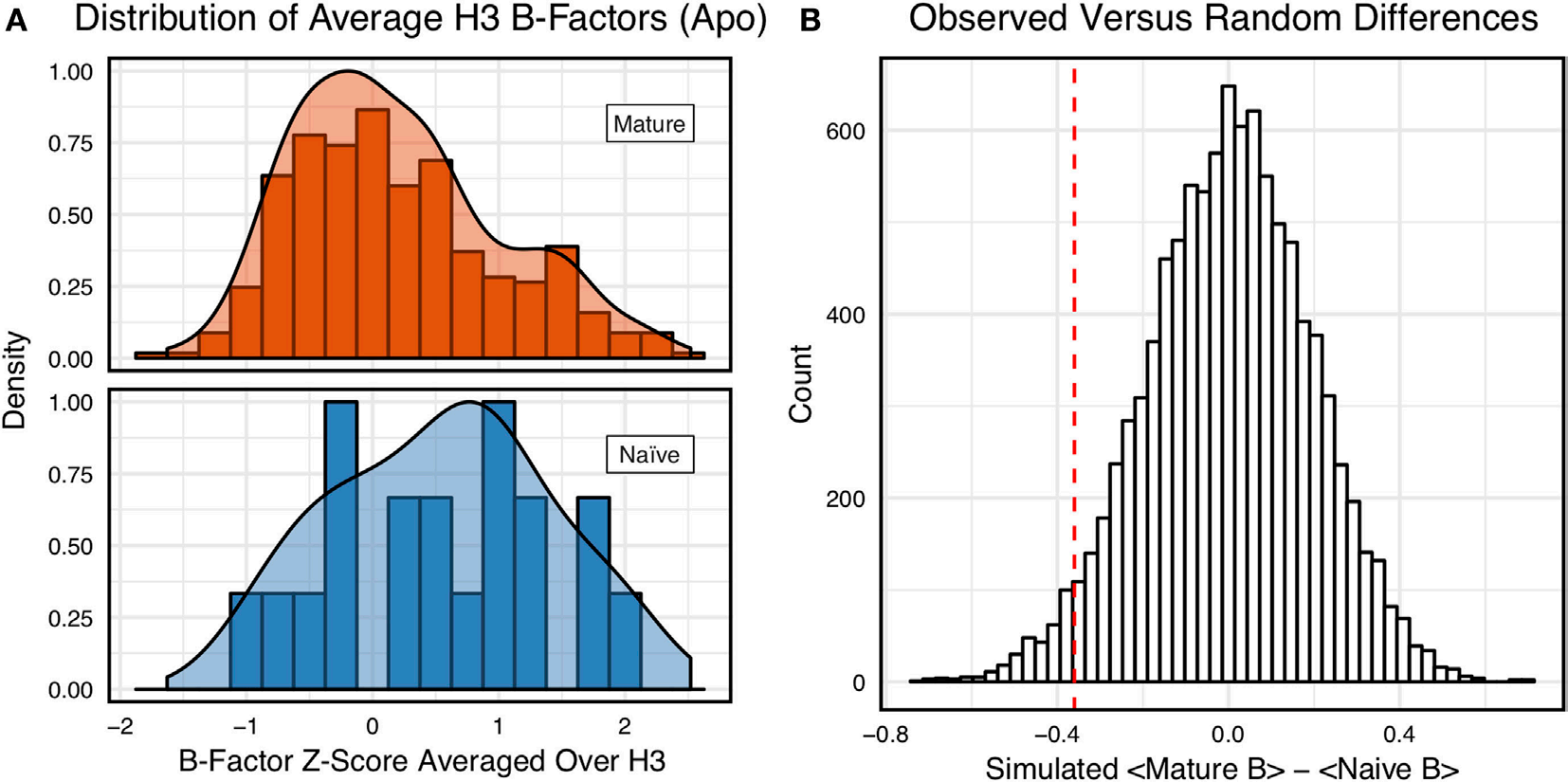
When considering only antigen-free crystal structures (to control for rigidification upon antigen biding), the difference between naïve and mature average CDR-H3 loop B-factor *z*-score distributions is small. **(A)** The distributions of CDR-H3 loop average B-factors are less distinct between the mature (orange) and naïve (blue) sets. Bars show binned counts in intervals of 0.25. Both the bars and smoothed densities are normalized so the maximum value is 1. A two-sample Kolmogorov–Smirnov test results in a *p*-value of 0.15 and *D* of 0.27, indicating that the null hypothesis of indistinguishable underlying distributions cannot be discarded. **(B)** The observed difference in distribution means (red line, dashed) is occasionally replicated in random resampling (white bars). When average CDR-H3 loop B-factor *z*-scores are pooled and randomly assigned to either a naïve or mature set, in the observed numbers (*N*_mature_ = 355 and *N*_naive_ = 18), the observed difference in means is matched or surpassed in 340 ± 20 out of 10,000 simulated differences.

However, we were concerned that the mixing of bound and unbound crystal structures would influence results, as we previously observed bound structures to have lower average B-factors (60). Furthermore, in the PDB-derived dataset, naïve antibodies were mostly crystallized in the unbound state (19 of 23), whereas mature antibodies were mostly co-crystallized with their cognate antigen (544 of 899). In conjunction, these two observations suggested that the high number of antigen-bound mature antibody crystal structures was the primary driver of the difference between naïve and mature B-factor *z*-scores. Thus, we compared the B-factor averages of unbound structures only and found that while the distributions appear to be distinct (Figure 4A), when the difference in distribution means is compared to a randomized set, 3.4 ± 0.2% of random differences are greater than or equal to the observed differences, and the distributions fail a two-sample KS test (*D* = 0.27, *p* = 0.15). Thus, the difference between naïve and mature antigen-free crystal structures does not appear significant.

**Figure 5:**
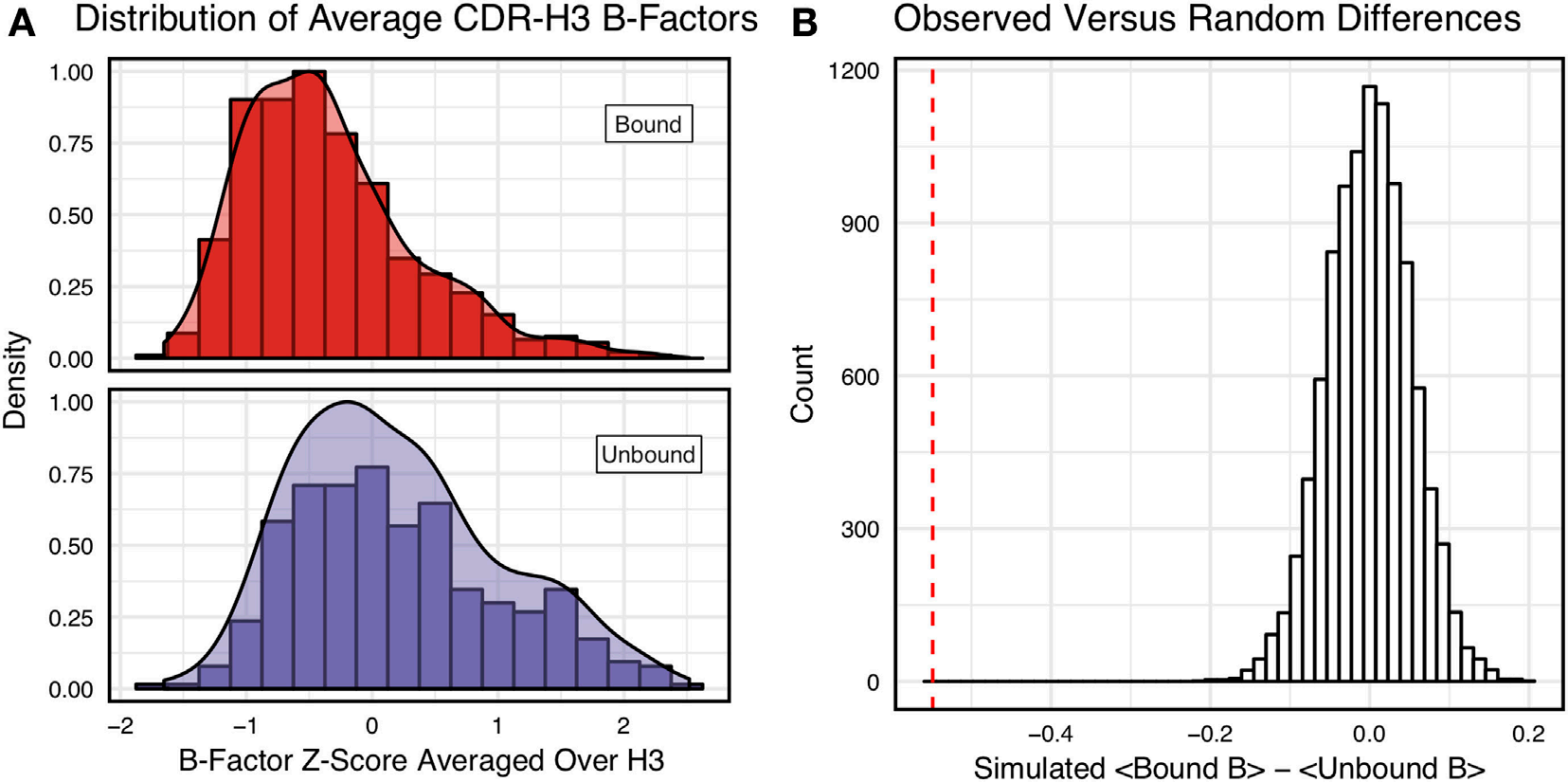
Antigen-bound and antigen-free distributions of B-factor *z*-scores are distinct. **(A)** Distributions of CDR-H3 loop average B-factors for the crystallographic set of antibodies are distinct for the antigen-bound (red) and antigen-free (purple) sets. Bound antibody CDR-H3 loops have lower B-factors than unbound, corresponding to more rigidity. Bars show binned counts in intervals of 0.25. Both the bars and smoothed densities are normalized so the maximum value is 1. Distributions appear distinct according to a two-sample Kolmogorov–Smirnov test with a *p*-value of 2.2E-16 and *D* of 0.31. **(B)** The observed difference in distribution means (red line, dashed) is never replicated in 10,000 attempts at random resampling (white bars). Simulated differences were acquired by randomly assigning values from both sets to either a naïve or mature set, in the observed numbers (N_bound_ = 546 and *N*_naive_ = 374), before computing the difference means.

As we conjectured, a significant difference was found between the bound and unbound distributions (Figure 5), with a two-sample KS test confirming the difference between the distributions (*D* = 0.31, *p* < 2.16E−16) and randomized testing never showing a difference in means as large as the observed difference. Additionally, we considered other possible origins of difference between the naïve and mature distributions that are not related to affinity maturation, including comparison across species, crystal structure resolutions, CDR-H3 loop lengths, and whether the CDR-H3 loop was at a crystal contact or not. We found none of these to have as clear of an effect on the distribution of B-factor averages as whether or not antigen was bound (Figures S5 and S6 in Supplementary Material). In summary, the distributions of B-factor *z*-score averages (Figures 3–5) suggest that both the naïve and mature antibody sets possess CDR-H3 loops of varying flexibility and that neither set is significantly more flexible or rigid than the other.

### Comparison of Mature to Naïve-Reverted Models Reveals Varying Rigidification across Matched Pairs

Having not observed consistent rigidification of the CDR-H3 loop in two large sets of antibodies, we postulated that rigidification was not a repertoire-wide phenomenon (i.e., all mature antibodies are not more rigid than all naïve antibodies), but it could still be plausible that matched pairs of naïve and mature antibodies would reveal rigidification.

To investigate this hypothesis, we selected 10 mature antibodies from our SAbDab set with CDR-H3 loops of length 10, a length for which loop modeling performs well (50, 61). We identified antibodies that had at least 5 (~97% sequence identity), but no more than 25 (~85% sequence identity), mutations when compared to the germline V-genes. To control for species, half of the selected antibodies were human and half were mouse. We reverted the mature antibody sequences to naïve using the germline sequences from the aligned V-genes, as described in the methods, and using germline J-genes from sequence alignments from IMGT/DomainGapAlign (47). The reverted sequences are reported in Section “Sequences Used to Model Naïve-Reverted Antibodies” in Supplemental Material. We then used RosettaAntibody to generate homology models for the naïve-reverted sequences. We analyzed the ensembles of the 10 lowest-energy homology models using FIRST-PG. To ensure fair comparison, we also used FIRST-PG to analyze homology model ensembles of the mature sequences. To provide an estimate for the accuracy of RosettaAntibody homology models, we computed RMSDs for the mature models using the known crystal structures and found all had sub-2-Å CDR-H3 loop backbone RMSD, calculated after alignment of the heavy chain FR, with 7 of 10 antibodies having sub-Å RMSD (Figures S7–S9 in Supplementary Material).

**Figure 6:**
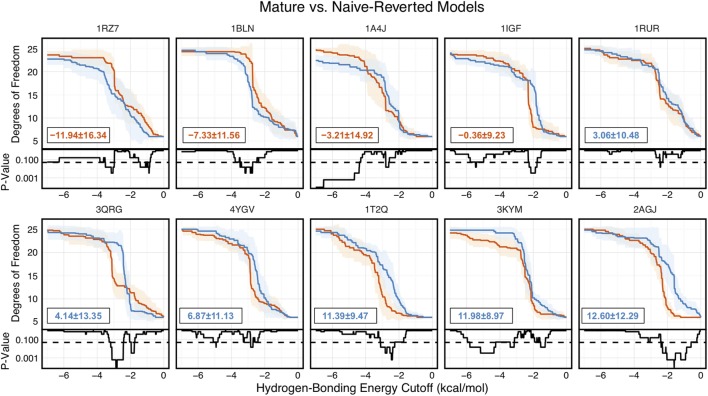
Floppy Inclusions and Rigid Substructure Topography-Pebble Game analysis of 10 RosettaAntibody-modeled mature/naïve-reverted antibody pairs (CDR-H3 loop length of 10 residues) shows that affinity maturation does not always result in CDR-H3 loop rigidification. Naïve values are colored blue, while mature values are color red. The difference between mature and naïve area under the curves (AUCs) is reported in the bottom left of each sub-figure, with a positive value indicate a more flexible naïve antibody. 4 out of the 10 cases have mature antibodies with AUC greater than their naïve counterparts. Subplots, below each main plot, show the *p*-value computed by a Kolmogorov–Smirnov test comparison of the naïve and mature degrees of freedom distributions for each hydrogen-bonding energy cutoff, with null hypothesis being that the distributions are the same and a dashed line indicating a *p*-value of 0.05.

Of the 10 naïve/mature antibody pairs we analyzed, 6 showed a decrease in flexibility and 4 showed an increase in flexibility upon affinity maturation (Figure 6). These 10 antibodies demonstrated the breadth of possible affinity maturation effects, from an expected flexibility decrease in antibody 2AGJ, with AUC decreasing by 9.34%, to the unexpected flexibility increase in antibody 1RZ7, with AUC increasing by 10.65%.

### Analysis of 48G7 Antibody

Having analyzed 1,911 models, 922 crystal structures, and 10 paired-reverted models, we had yet to observe a consistent difference in CDR-H3 loop flexibility between naïve and mature antibodies, as previously reported in literature. Thus, we turned to three previously studied antibodies with known crystal structures and measured CDR-H3 loop flexibility. These are (1) the esterolytic antibody 48G7 (16, 32, 33, 35), (2) the anti-fluorescein antibody 4-4-20 (23, 26–28, 31, 33), and (3) a broadly neutralizing influenza virus antibody (22). For all three antibodies, the effects of affinity maturation on CDR-H3 loop flexibility have been previously studied by both experiment and simulation, allowing comparison with our results. For brevity, we presently discuss the 48G7 antibody here and full results for all antibodies are available in the Supplementary Material.

**Figure 7:**
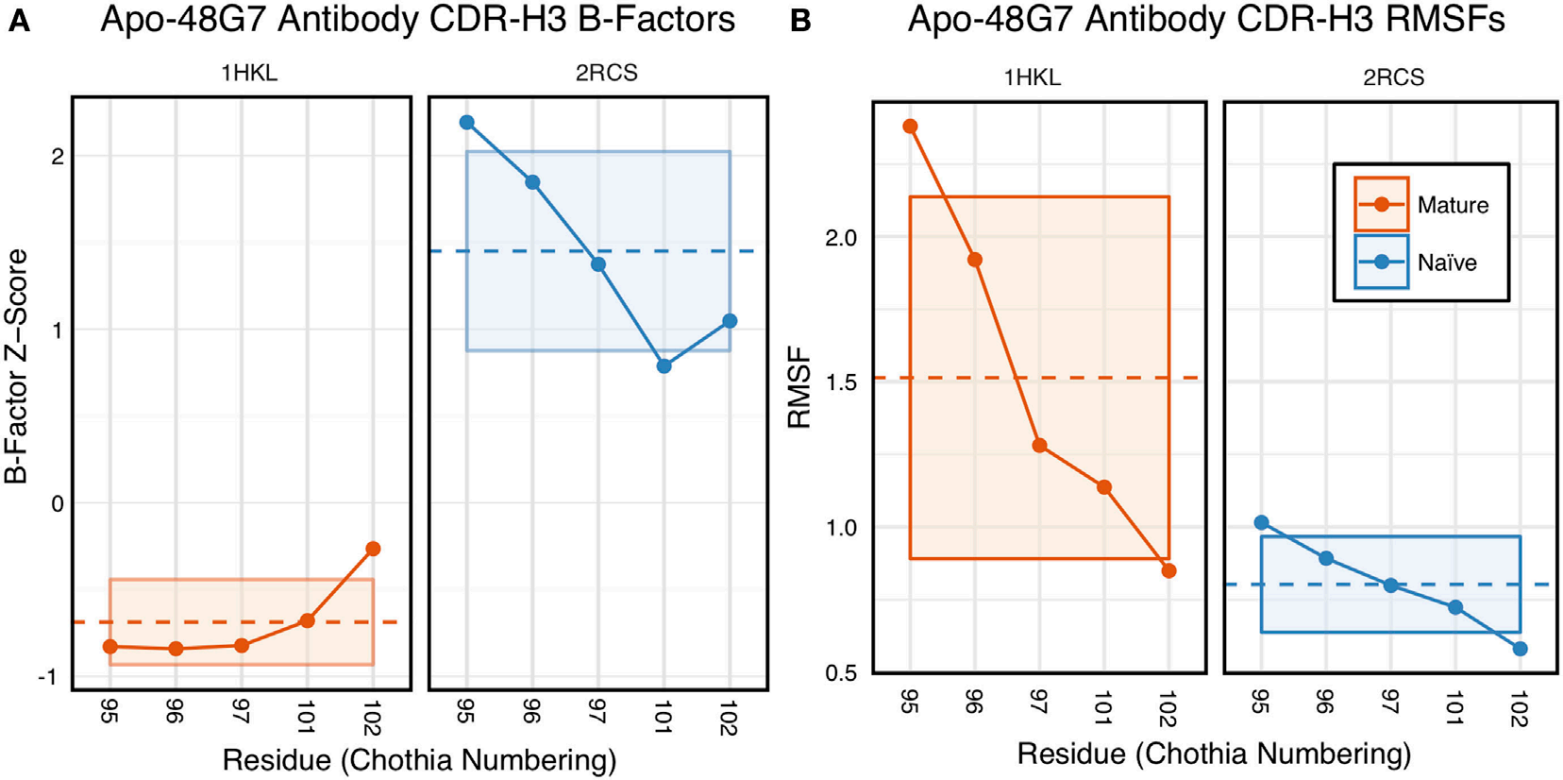
Analysis of catalytic antibody 48G7 by CDR-H3 loop B-factors and root-mean-square fluctuations (RMSFs) shows conflicting results. **(A)** Comparison of normalized B-factor values for the CDR-H3 loop of the 48G7 antibody in crystal structures of the unbound naïve (dark blue) and mature (dark orange) antibodies reveals a more rigidity in the mature antibody. The dashed line indicates the average value and is outlined by a box defined by the average plus-or-minus the SD. **(B)** Comparison of CDR-H3 loop RMSFs for the molecular dynamics simulations of the naïve and mature 48G7 antibodies shows the opposite.

The 48G7 antibody was first studied through crystallography, with structures capturing the bound (holo) and unbound (apo) states of both the naïve and mature antibody (16). Comparison between the naïve and mature CDR loop motions from the free to the bound state revealed minor changes, with the mature CDR-H3 loop being slightly more rigid and moving an Angstrom less than the naïve upon antigen binding (Figures S10 and S11 in Supplementary Material). For each of the four crystal structures, we extracted B-factors and computed B-factor *z*-scores for the CDR-H3 loop, measuring the distance from the B-factor mean in SDs. B-factor *z*-scores for the CDR-H3 loop of apo-48G7 are shown in Figure 7A. The mature antibody has lower B-factors than the naïve antibody throughout the entire CDR-H3 loop. This observation also holds for the holo-48G7 antibody structures as well (Figure S12 in Supplementary Material). Table S2 in Supplementary Material summarizes B-factors, averaged over the whole CDR-H3 loop. These B-factor results agree with the prior crystallographic observations.

Follow-up studies on 48G7 used MD simulations to assess flexibility. Briefly, 500 ps short MD simulations of the naïve and mature antibodies in the presence of antigen with an explicit solvent model (TIP3P) found the CDR-H3 loop to be more flexible in the naïve than in the mature antibody by comparison of RMSFs (30), but 15 ns MD simulations of the naïve and mature antibodies in the absence of antigen with an implicit solvent model (GB/SA) found no difference between the two, again by comparison of RMSFs (32). Another study based on an elastic network model also suggested that, in the absence of antigen, the fluctuations of the naïve and mature 48G7 were similar, but their binding mechanisms could differ depending on response to antigen binding; the naïve antibody shows a discrete conformational change induced by antigen, whereas the mature antibody shows lock-and-key binding (62). Due to the contentious nature of these results, we ran 200 ns MD simulations for the 48G7 naïve and mature antibodies in the absence of antigen with an explicit solvent model (TIP3P). We measured both RMSDs and RMSFs for the Cα atoms along the CDR-H3 loop and computed the difference between the naïve and mature antibodies (Table S2 in Supplementary Material). Figure 7B shows that the CDR-H3 loop RMSFs are consistently greater for the mature than the naïve 48G7 antibody.

**Figure 8:**
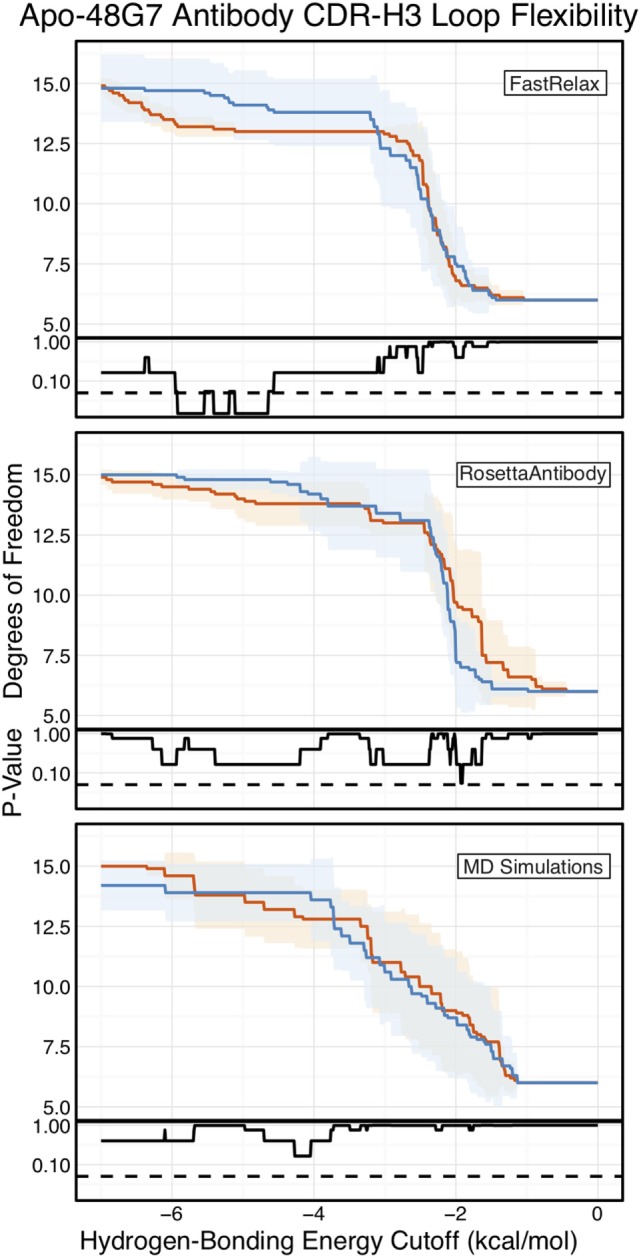
Floppy inclusions and rigid substructure topography-Pebble Game (FIRST-PG) analysis of naïve (dark blue) and mature (dark orange) 48G7 antibodies using either Rosetta FastRelax-, RosettaAntibody-, or molecular dynamics (MD)-generated 10-member ensembles does not show a difference between the naïve and mature antibodies. FIRST-PG analysis calculates the degrees of freedom (DOFs) of CDR-H3 loop as a function of hydrogen-bonding energy cutoff. Subplots, below each main plot, show the *p*-value computed by a Kolmogorov–Smirnov test comparison of the naïve and mature DOF distributions for each hydrogen-bonding energy cutoff, with null hypothesis being that the distributions are the same and a dashed line indicating a *p*-value of 0.05. While the FastRelax ensembles appear distinct in the range of −6 to −3 kcal/mol, the naïve and mature are indistinguishable for both the RosettaAntibody and MD ensembles.

Finally, as we have done through this study, we used FIRST-PG to measure CDR-H3 loop flexibility. To limit the effects of crystal structure artifacts on FIRST-PG analysis, we used an ensemble of 10 representative structures, derived by clustering trajectory frames and selecting 10 structurally distinct cluster medians from the MD simulations, similar to a previous flexibility study for this antibody (33). The CDR-H3 loop flexibility of apo-48G7, as determined by FIRST-PG analysis of MD ensembles is shown in Figure 8. The FIRST-PG analysis showed no significant difference between the mature and naïve antibodies.

In addition to using MD simulations to generate ensembles, we used ensembles generated by RosettaAntibody and Rosetta FastRelax, permitting direct comparison. The CDR-H3 loop flexibility of apo-48G7, determined by FIRST-PG analysis of FastRelax and RosettaAntibody ensembles, is shown in Figure 8. The curves from FastRelax and the MD simulation are similar for low-energy cutoffs (e.g., in the range of 0.0 to −3.0 kcal/mol), with the naïve and mature DOFs being the same. These curves diverge at higher energy cutoffs, where the FastRelax curve shows a more flexible naïve antibody and the MD curve does not. The curve from RosettaAntibody ensembles differs from the two and shows a more flexible mature antibody at low-energy cutoffs and a more flexible naïve at high-energy cutoffs. For less visual and more quantitative comparisons, we computed the AUC of the DOF vs. hydrogen-bonding energy cutoff plots (Table S2 in Supplementary Material). We find the AUC is only slightly greater for naïve than mature antibodies in the FastRelax and RosettaAntibody ensembles, with the naïve AUC reducing by only 3.9 and 0.2%, respectively, upon maturation. MD ensembles show the opposite outcome, with the mature antibody having 1.3% greater AUC than the naïve.

Further validation was carried out on two other previously studied antibodies and reported in the Table S2 and Figures S12 and S13 in Supplementary Material. For the 4-4-20 antibody, antigen-bound structures were compared and the average mature B-factors were within a SD of the naïve. For the influenza antibody, average B-factors were compared between an unbound naïve and a bound mature crystal structure, showing significant rigidification. However, results are conflated due to the lack of unbound crystal structures, as in bound structures antibody–antigen contacts artificially increase rigidity of the CDR-H3 loop. In contrast to B-factor analyses, FIRST-PG analyses yielded mixed results for these two antibodies. The 4-4-20 antibody was found to become more flexible upon maturation by FIRST-PG analysis of all, but Rosetta KIC ensembles. The influenza antibody was found to become more rigid upon mature by FIRST-PG analysis of all, but Rosetta FastRelax ensembles. Finally, we analyzed RMSDs and RMSFs from MD simulations and found that the mature 4-4-20 antibody has higher CDR-H3 loop RMSD, but lower RMSF, values than the naïve while the mature influenza antibody was found to have lower values for both (Table S2 in Supplementary Material). As with our repertoire analysis, we do not see consistent rigidification in previously studied antibodies. We consider the significance of this result and compare our analysis in detail to past analyses of flexibility in Section “Discussion.”

## Discussion

### The Varying Effects of Affinity Maturation on CDR-H3 Flexibility

Affinity maturation, through a series of somatic hypermutation events and selection processes, can evolve a low-affinity, naïve antibody to bind an antigen with both high affinity and specificity (63). Elucidating the affinity maturation process is desirable to understand molecular evolution, develop antibody engineering methods, and guide vaccine development (64). Past studies have suggested that, with few exceptions (29, 65, 66), naïve antibodies are highly flexible and maturation leads to improved affinity and specificity through the optimization and rigidification of the antibody paratope, and in particular the CDR-H3 loop (22, 27, 28, 31–33). However, these studies have been limited, often focusing on a single antibody and assessing flexibility indirectly. We sought to test the generalizability of the rigidification-upon-maturation hypothesis. We were enabled by the large number of antibody structures in the PDB, homology models generated from high-throughput repertoire sequencing data, and the FIRST-PG method for rapid structural flexibility calculation to ask whether affinity maturation leads to CDR-H3 loop rigidification.

Unexpectedly, in a comparison of flexibility of repertoires, our data show little difference between naïve and mature antibodies: FIRST-PG calculations showed no difference for RosettaAntibody homology model ensembles of the most common naïve and mature antibodies in human peripheral blood cells. The same calculations showed no difference in CDR-H3 loop DOFs of crystal structures under two different refinement schemes (FastRelax and KIC). After accounting for the presence/absence of antigen, CDR-H3 loop B-factor distributions were similar for both mature and naïve antibody crystal structures. These results indicate that rigidification of the CDR-H3 loop does not always occur upon affinity maturation.

Since our observations did not indicate clear rigidification over two sets of antibodies, we considered the following possibilities: (1) comparison of different length CDR-H3 loops was unfair because longer loops are inherently more flexible, (2) comparison of different antibodies was unfair because different combinations of gene segments and V_H_–V_L_ pairs will result in different flexibilities, (3) mutations within CDR-H3 loop, which we could not identify for the PDB set because of the difficulty in D/J-gene alignments, may have modulated flexibilities of CDR-H3, (4) inaccuracies in the computational methods could preclude observation of rigidification, and (5) FIRST-PG-measured backbone DOFs are not a good measure of flexibility. To address the first concern, we analyzed loops of consistent length *via* B-factor and FIRST-PG (Figures 1B and 2B; Figures S4 and S5 in Supplementary Material). We found that, according to KS testing and when accounting for the presence/absence of antigen, B-factor distributions were not distinct for naïve and mature sets of antibodies with same length CDR-H3 loops (length 10 for the crystallographic set and 12 for the repertoire model set). We also found that FIRST-PG DOFs AUC values of the naïve and mature sets of antibodies with the same length CDR-H3 loops were within a SD for RosettaAntibody, FastRelax, and KIC ensembles. So, even when accounting for length, mature antibodies are not significantly more rigid than naïve ones.

To address the concern that comparison of sets of antibodies originating from different V_H_ and V_L_ genes is unfair, we analyzed mature/naïve antibody pairs that had been previously studied and mature/naïve-reverted pairs that we generated with RosettaAntibody and analyzed by FIRST-PG (Figures 6–8; Table S2 in Supplementary Material). We found that CDR-H3 loop B-factors did not always indicate rigidification upon maturation and for the 7G12 antibody we observed the reverse effect (Figure S14 in Supplementary Material). We also found that mature antibodies did not always become more flexible upon naïve reversion, but instead displayed a breadth of behaviors (Figure 6). So, when analyzing matched naïve/mature pairs, we do not see consistent rigidification upon maturation.

Our analysis of previously studied naïve/mature antibody pairs coupled with the earlier repertoire analysis should alleviate concerns that our flexibility results for the PDB set were strongly affected by our inability to align D/J-gene segments, and thus consider mutations in the CDR-H3 loop. The previously studied pairs included CDR-H3 mutations and the repertoire set had antibody sequences determined by Illumina MiSeq sequencing with naïve/mature status assigned by the absence/presence of the CD27 cell-surface receptor. In both cases, the naïve and mature sequences were determined through the entire Fv, and flexibility analysis still revealed mixed results.

Finally, to address the concern that RosettaAntibody models may not be accurate enough to be useful for FIRST-PG calculations, we tested FIRST-PG on a range of structural ensembles with varying deviation from the crystal structure. We found no difference in the naïve vs. mature antibody CDR-H3 loop AUC of the FIRST-PG results, regardless of the ensemble generation method used (compare Figure 2; Figure S4 in Supplementary Material). We also determined flexibility through alternative measures, such as crystal structure B-factors and RMSFs in MD simulations. For both, affinity maturation was not found to have a consistent, rigidifying effect. Thus, even if model inaccuracies confound analysis, other data support the same hypothesis.

### Comparison with Prior Results

Our analysis included several antibodies that have been the subject of previous flexibility studies, permitting a direct comparison (Table S4 in Supplementary Material summarizes past studies). One of the most studied antibodies is the anti-fluorescein antibody, 4-4-20. Spectroscopic experiments measuring the response of a fluorescent probe (fluorescein) and MD simulations measuring Cα atom fluctuations suggested that somatic mutations restrict conformational fluctuations in the mature antibody (26, 28, 31). Our analysis of 4-4-20 was not as clear: we observed no significant difference in naïve vs. mature CDR-H3 loop crystallographic B-factors (Figure S12 in Supplementary Material) and found the mature antibody to be more rigid in FIRST-PG calculations only in the −2.0 to –0.0 kcal/mol range of hydrogen-bonding energy cutoffs (Figure S13 in Supplementary Material). Similar mixed results were observed by Li et al. (33) who used a Distance Constraint Model (DCM) to analyze flexibility in an ensemble of 4-4-20 conformations drawn from MD simulations. They found increases in structural rigidity of the CDR-H3 loop, as determined by the DCM, occurred upon affinity maturation, but these increases did not correspond to decreases in dynamic conformational fluctuations, as determined by RMSFs from MD simulations. Further studies artificially matured 4-4-20 by directed evolution, resulting in a femtomolar-affinity antibody, 4M5.3 (67), but the crystal structures of 4M5.3 and 4-4-20 were almost identical (the reported backbone RMSD is 0.60 Å) and thermodynamic measurements suggested that the affinity improvement was achieved primarily through the enthalpic interactions with subtle conformational changes (68). This observation was contradicted by Fukunishi et al. (69), who performed steered MD simulations to analyze the effects of the mutations on the flexibility of 4-4-20 and 4M5.3. By applying external pulling forces between the antibodies and the antigen along a reaction coordinate, they quantified the interactions and showed that, during the simulations, fluctuations of the antibody, especially the CDR-H3 loop, and of the antigen were indeed larger in 4-4-20 than in the more matured antibody, 4M5.3 (69). Thus, there is some variation not only in our results, but also in the literature as to the effects of affinity maturation on 4-4-20.

Another set of well-studied antibodies are the four catalytic antibodies: 48G7, 7G12, 28B4, and AZ-28. In fact, the first crystallography studies to suggest rigidification of the CDR-H3 loop as a consequence of affinity maturation were performed on 48G7. Wedemayer et al. observed larger structural rearrangements upon antigen binding in the CDR-H3 loop for the naïve antibody than the mature antibody (Figures S10 and S11 in Supplementary Material) (16). Crystallization of the naïve unbound, naïve bound, mature unbound, and mature bound states for 7G12, 28B4, and AZ-28 revealed similar results (18, 19). Additionally, MD simulations of the four catalytic antibodies in implicit solvent were used to calculate CDR Cα atom B-factors (32). Wong et al. showed a decrease in mature CDR-H3 loop B-factors in three cases (7G12, 28B4, and AZ-28), whereas no significant difference was observed for 48G7 (see Figure 2 in Wong et al.). Furthermore, for 48G7, Li et al. used MD simulation to generate structural ensembles and DCM analysis to determine flexibility. They found that the mature CDR-H3 loop is more rigid than the naïve, according to DCM, but used an unusual loop definition that included five additional flanking residues (see Figure 1 in Li et al.), making comparison challenging (longer loops will be inherently more flexible), and they observed increases in the mature CDR-H3 loop RMSFs (see Figure 8 in Li et al.) (33). Our analysis of CDR-H3 loop B-factors showed rigidification upon maturation for some of the 48G7 and 28B4 crystal structures (Figure 7; Figure S14 in Supplementary Material), but not for 7G12 and AZ-28 structures (Figures S14 and S15 in Supplementary Material). FIRST-PG analysis of FastRelax, RosettaAntibody, and MD ensembles for 48G7 showed slight to no rigidification (Figure 8). Additionally, RMSFs from MD simulations for 48G7 showed higher values for the mature loop, contrary to the expectation that it is more rigid. Our mixed results for the effects of affinity maturation on 48G7 are consistent with literature, but there is variation between our results and the literature as to the effects of affinity maturation on the other catalytic antibodies.

Finally, Schmidt et al. used X-ray crystallography, MD simulations, and thermodynamics measurements to investigate how somatic mutations affected the binding mechanism of anti-influenza antibodies (22). They identified three mature antibodies, their unmutated common ancestor (UCA), and a common intermediate, all derived from a subject immunized with an influenza vaccine. The affinities of the mature antibodies were about 200-fold better than the UCA. MD simulations of the UCA and the mature antibodies showed that CDR-H3 loop of the UCA could sample more diverse conformations than the mature antibodies, whose CDR-H3 loop sampled only conformations optimal for antigen binding, supporting the hypothesis that somatic mutations rigidify antibody structures. In another study by the same group (70), further MD simulations were performed on the same systems, showing that, although many somatic mutations typically accumulate in broadly neutralizing antibodies during maturation, only a handful of mutations substantially stabilize CDR-H3 loop and hence enhance the affinity of the antibodies for antigen. In our studies, all the results (Figures S12 and S13 and Table S2 in Supplementary Material) for the anti-influenza antibody, except FIRST-PG flexibility calculations for the Rosetta FastRelax ensemble, show rigidification of the CDR-H3 loop as an effect of affinity maturation and agree with the detailed analysis of Schmidt et al.

For the three antibody families we analyzed in detail, we observed mixed effects of affinity maturation on two (catalytic antibodies and 4-4-20) and clear rigidification in one (anti-influenza antibody). For the two with mixed results, we note that past work has also shown conflicting results. We interpret these results as supportive of our repertoire-wide analysis that affinity maturation does not always rigidify the CDR-H3 loop.

### Biophysical Properties Underlying Antibody Binding

Why is antibody CDR-H3 loop rigidification not a consistent result of affinity maturation? Consider the process of affinity maturation, which selects for antibody–antigen binding and against interactions with self or damaged antibodies (i.e., when deleterious mutations are introduced by activation-induced cytidine deaminase) (71). Under these selection pressures, what is the benefit of CDR-H3 loop rigidification? Loop rigidification can only decrease the protein-entropy cost for antibody–antigen binding, having ostensibly no effect on enthalpy and solvent entropy of binding, and self-interactions. If CDR-H3 loop rigidification is just one of many biophysical mechanisms that can be selected for during affinity maturation, then we do not expect to observe it consistently, in line with our results.

What are the other possible mechanisms then? Collectively, studies have shown that improved antibody affinity and specificity for antigen can be achieved by introducing additional interfacial interactions, including hydrogen bonds, salt bridges, and van der Waals contacts (16, 72–74); increasing the buried surface area, either polar or apolar, depending on the antigen (20); and improving interface shape complementarity (20, 75), in addition to rigidification of the paratope (22). A detailed review on the structural basis of antibody affinity maturation, by Mishra and Mariuzza, can be found in this research topic (76).

An interesting consequence of the biological antibody selection process is the anti-hapten antibody, SPE7 (77). For SPE7, mutations leading to multi-specificity or promiscuity were beneficial—antibodies are multivalent, so an antibody capable of binding multiple antigens with intermediate affinity can gain an effective advantage through cooperative binding over an antibody capable of binding only one antigen. Crystal structures of SPE7 with different antigens and in its apo-state demonstrated that SPE7 can assume different conformations. Motivated by these observations, Wang et al. exploited MD simulations to investigate the binding mechanisms of SPE7 (78). The MD simulations and subsequent analyses suggested that multi-specific antigen binding is mediated by a combined mechanism of conformer selection and induced fit. Similar behavior, where the mature antibody is more flexible than the naïve has been observed for an antibody that recognizes the tumor-associated ganglioside GD2 (79). Such antibodies could not have arisen if CDR-H3 loop rigidification were a consistent result of affinity maturation.

## Conclusion

We have conducted the largest-scale flexibility study of antibody CDR-H3 loops, analyzing 922 crystal structures and 1,911 homology models. We used B-factors and FIRST-PG to assess flexibility. We sought to identify the effects of affinity maturation on CDR-H3 loop flexibility, expecting the CDR-H3 loop to rigidify. We found that there were no differences in the CDR-H3 loop B-factor distributions or FIRST-PG DOFs for naïve vs. mature antibody crystal structures and in the CDR-H3 FIRST-PG DOFs for homology models of repertoires of naïve and mature antibodies. These findings suggest that there is no general difference between naïve and mature antibody CDR-H3 loop flexibility in repertoires of naïve and mature antibodies. However, we observed rigidification of the CDR-H3 loop for some, but not all, antibodies when the mature antibodies were compared directly to their germline predecessors. So, we conclude that increased rigidity occurs alongside other affinity increasing changes, such as improved interfacial interactions, increased buried surface area, and improved shape complementarity.

Further work must be done to address the issues observed here, i.e., inconsistent results across the different methods are used to measure flexibility. One possible route is to explore experimental methods that directly measure protein dynamics across several timescales, and use them to study a relatively large (more than one or two antibodies) and diverse (e.g., from different source organisms or capable of binding different antigens) set of antibodies. For example, HDX-MS is capable of identifying protein regions with dynamics on timescales from milliseconds to days, has been previously used to study antibody dynamics, and has been correlated to FIRST-PG (29, 41, 80).

Finally, we note the need for more rapid and accurate antibody modeling methods. With the advent of high-throughput sequencing, there now exits a plethora of antibody sequence data, but little structural data. Accurate modeling can overcome the lack of high-throughput structure determination method and provide crucial structural data. These structures can then be used to address scientific questions on a larger scale than before, on the scale of the human antibody repertoire.

## Author Contributions

JJ, AS, DK, and JG designed the research. JJ, AS, DK, and NT performed the research. JJ, AS, and DK analyzed the data. JJ, AS, DK, NT, NK, KT, and JG wrote the paper.

## Conflict of Interest Statement

The authors declare that the research was conducted in the absence of any commercial or financial relationships that could be construed as a potential conflict of interest. The reviewer OL declared a past co-authorship with several of the authors (DK JG) to the handling editor.
