# Supplementary material for: Repertoire Analysis of Antibody CDR-H3 Loops Suggests Affinity Maturation Does Not Typically Result in Rigidification
Source: Front Immunol. 2018 Mar 2;9:413. doi: 10.3389/fimmu.2018.00413 (PMC5840193; doi:10.3389/fimmu.2018.00413)
Supplement: Supplementary file 1 [file data_sheet_1.pdf]

Supplementary Material

Repertoire analysis of antibody CDR-H3 loops suggests affinity maturation does not typically result in rigidification

Jeliazko R. Jeliazkov†, Adnan Sljoka†,*, Daisuke Kuroda, Nobuyuki Tsuchimura, Naoki Katoh, Kouhei Tsumoto, Jeffrey J. Gray*

*** Correspondence:** adnanslj@gmail.com or jgray@jhu.edu

Table of Contents

1 Supplementary Data 2

1.1 Supplementary Command Lines.......... 2

1.1.1 Rosetta FastRelax[1–3] 2

1.1.2 Rosetta KIC[4,5] 2

1.1.3 RosettaAntibody Homology Modeling[6,7] 3

1.2 Sequences Used to Model Naïve-Reverted Antibodies 4

1.3 Comparison of Flexibility Calculations Across Ensemble Generation Methods 8

2 Supplementary Figures and Tables 9

2.1 Supplementary Figures 9

2.2 Supplementary Tables.......... 25

3 References 33

1 Supplementary Data

1.1 Supplementary Command Lines

Rosetta version 2017.26-dev59567 was used for all simulations.

1.1.1 Rosetta FastRelax[1–3]

Antibody Fv regions were relaxed with the following command and options:

```
/path/to/relax.linuxgccrelease -l pdb.list \
    -ex1 \
    -ex2 \
    -use_input_sc \
    -beta \
    -nstruct 10
```

1.1.2 Rosetta KIC[4,5]

Antibody Fv regions had their CDR-H3 loop remodeled and relative V_H–V_L orientation resampled with the following command and options:

```
/path/to/antibody_H3.linuxgccrelease -l pdb.list \
-ex1 \
-ex2 \
-nstruct 10 @abH3.flags
```

where, `abH3.flags` is a file containing the following additional options:

```
-antibody::remodel              perturb_kic
-antibody::snugfit              true
-antibody::refine               refine_kic
-antibody::cter_insert          false
-antibody::flank_residue_min    true
-antibody::bad_nter             false
-antibody::h3_filter            false
-antibody::h3_filter_tolerance  5

-extrachi_cutoff 0
-loops:legacy_kic false
-loops:kic_min_after_repack true
-loops:kic_omega_sampling
-loops:allow_omega_move true
-kic_bump_overlap_factor 0.36
-loops:ramp_fa_rep
-loops:ramp_rama
-loops:refine_outer_cycles 2
-loops:max_inner_cycles 20
```

1.1.3 RosettaAntibody Homology Modeling[6,7]

Antibody Fv homology models were generate with RosettaAntibody in three steps: (1) assembly of the homologous components, (2) FastRelax of the grafted model, (3) CDR-H3 loop modeling and V_H–V_L docking.

Homologous components were selected and assembled with the following command and options:

```
/path/to/antibody.macosclangrelease -fasta pdb.fasta \
-antibody:n_multi_templates 1 \
-antibody:no_relax
```

The resulting "model-0.pdb" was the relaxed with constraints by:

```
relax.macosclangrelease -s model-0.pdb \
-flip_HNQ \
-no_optH false \
-relax:fast \
-relax:constrain_relax_to_start_coords \
-relax:ramp_constraints false \
-use_input_sc \
-ex1 \
-ex2 \
-nstruct 1
```

Finally, CDR-H3 loop modeling and docking of VH–VL was done by:

```
/path/to/antibody_H3.linuxgccrelease -s grafting/model-0_0001.pdb \
-nstruct 1000 @abH3.flags
```

with the following `abH3.flags`:

```
-antibody::remodel              perturb_kic
-antibody::snugfit              true
-antibody::refine               refine_kic
-antibody::cter_insert          false
-antibody::flank_residue_min    true
-antibody::bad_nter             false
-antibody::h3_filter            false
-antibody::h3_filter_tolerance  5
-antibody:constrain_vlvh_qq

-ex1
-ex2
-extrachi_cutoff 0

-loops:legacy_kic false
-loops:kic_min_after_repack true
-loops:kic_omega_sampling
```

```
-loops:allow_omega_move true
-kic_bump_overlap_factor 0.36
-loops:ramp_fa_rep
-loops:ramp_rama
-loops:refine_outer_cycles 5
```

1.2 Sequences Used to Model Naïve-Reverted Antibodies

Mature sequences were aligned to germline V-genes as described in the methods. Additionally, sequences were aligned to germline J-genes using IMGT/DomainGapAlign, which yields germline alignments for both V- and J-genes. For example, the alignment of the variable region of 1T2Q can be extracted from: http://www.imgt.org/3Dstructure-DB/cgi/details.cgi?pdbcode=1t2q. The germline sequence was used when possible. The alignments are shown below with the mature sequence above and naive below.

1A4J Heavy

```
QVQLLESGPELKKPGETVKISCKASGYTFTNYGMNWVKQAPGKGLKWMGWINTYTGEPTYADDFKGRFAFSLETSASTAY
|*||**||||||||||||||||||||||||||||||||||||||||||||||||||||||||||||||||||||||||||
QIQLVQSGPELKKPGETVKISCKASGYTFTNYGMNWVKQAPGKGLKWMGWINTYTGEPTYADDFKGRFAFSLETSASTAY

LQINNLKNEDTATYFCVQAERLRRTFDYWGAGTTVTVS
||||||||||||||||**|||| **||*||||||||||
LQINNLKNEDTATYFCARAERL-YWFDVWGAGTTVTVS
```

1A4J Light

```
ELVMTQTPLSLPVSLGDQASISCRSSQSLVHSNGNTYLHWYLQKPGQSPKLLIYKVSNRFSGVPDRFSGSGSGTDFTLKI
**||||||||||||||||||||||||||||||||||||||||||||||||||||||||||||||||||||||||||||||
DVVMTQTPLSLPVSLGDQASISCRSSQSLVHSNGNTYLHWYLQKPGQSPKLLIYKVSNRFSGVPDRFSGSGSGTDFTLKI

SRVEAEDLGVYFCSQSTHVPPTFGGGTKLEIKR-
|||||||||||||||||||||||||||||||*|
SRVEAEDLGVYFCSQSTHVPPTFGGGTKLEINRA
```

1BLN Heavy

```
EVILVESGGGLVKPGGSLKLSCAASGFTFSSYTMSWVRQTPEKRLEWVATISSGGGNTYYPDSVKGRFTISRDNAKNNLY
||*|||||||||||||||||||||||||||||||||||||||||||||||||||||||||||||||||||||||||||||
EVMLVESGGGLVKPGGSLKLSCAASGFTFSSYTMSWVRQTPEKRLEWVATISSGGGNTYYPDSVKGRFTISRDNAKNNLY

LQMSSLRSEDTALYYCARYYRYEAWFASWGQGTLVTVS
|||||||||||||||||||||||||||*||||||||||
LQMSSLRSEDTALYYCARYYRYEAWFAYWGQGTLVTVS
```

1BLN Light

```
DVLMTQTPVSLSVSLGDQASISCRSSQSIVHSTGNTYLEWYLQKPGQSPKLLIYKISNRFSGVPDRFSGSGSGTDFTLKI
||||||||*||*|||||||||||||||||||||*||||||||||||||||||||||*|||||||||||||||||||||||
DVLMTQTPLSLPVSLGDQASISCRSSQSIVHSNGNTYLEWYLQKPGQSPKLLIYKVSNRFSGVPDRFSGSGSGTDFTLKI

SRVEAEDLGVYYCFQASHAPRTFGGGTKLEIKR-
|||||||||||||||*||*|*||||||||||||
SRVEAEDLGVYYCFQGSHVPWTFGGGTKLEIKRA
```

1IGF Heavy

```
EVQLVESGGDLVKPGGSLKLSCAASGFTFSRCAMSWVRQTPEKRLEWVAGISSGGSYTFYPDTVKGRFIISRNNARNTLS
|||||||||*||||||||||||||||||||**|||||||||||||||||*||||||||*|||*|||||*|||*||*|||*
EVQLVESGGGLVKPGGSLKLSCAASGFTFSSYAMSWVRQTPEKRLEWVATISSGGSYTYYPDSVKGRFTISRDNAKNTLY

LQMSSLRSEDTAIYYCTRYSSDPFYFDYWGQGTTLTVS
||||||||||||*|||*|||||||||||||||||||||
LQMSSLRSEDTAMYYCARYSSDPFYFDYWGQGTTLTVS
```

1IGF Light

```
DVLMTQTPLSLPVSLGDQASISCRSNQTILLSDGDTYLEWYLQKPGQSPKLLIYKVSNRFSGVPDRFSGSGSGTDFTLKI
|||||||||||||||||||||||||*|*|**|*|*|||||||||||||||||||||||||||||||||||||||||||||
DVLMTQTPLSLPVSLGDQASISCRSSQSIVHSNGNTYLEWYLQKPGQSPKLLIYKVSNRFSGVPDRFSGSGSGTDFTLKI

SRVEAEDLGVYYCFQGSHVPPTFGGGTKLEIKR-
|||||||||||||||||||||||||||||||||
SRVEAEDLGVYYCFQGSHVPPTFGGGTKLEIKRA
```

1RUR Heavy

```
EVQLEESGPELVRPGTSVKISCKASGYTFTNYWLGWVKQRPGHGFEWIGDIYPGGVYTTNNEKFRGKAILTADTSSSTAY
*|||**||*||||||||||*||||*||||||||*||||||||||*||||||||||*||**||||*|||*|||||||||||
QVQLQQSGAELVRPGTSVKMSCKAAGYTFTNYWIGWVKQRPGHGLEWIGDIYPGGGYTNYNEKFKGKATLTADTSSSTAY

MQLSSLTSEDSAVYFCARAGGYYTGGDYWGQGTSVTVS
||||||||||||*|*|||||||||**||||||||*|||
MQLSSLTSEDSAIYYCARAGGYYTYFDYWGQGTSLTVS
```

1RUR Light

```
DIVLTQAAFSNPVTLGASASISCRSSKSLLNSNGIIHMYWYLQKPGQSPQLLIYQMSKLASGAPDRFSGSGSGTDFTLRI
|||*||||||||||||*|||||||||||||*||||***|||||||||||||||||||*||||*|||||*|||||||||||
DIVMTQAAFSNPVTLGTSASISCRSSKSLLHSNGITYLYWYLQKPGQSPQLLIYQMSNLASGVPDRFSSSGSGTDFTLRI

SRVEAEDVGVYYCAQNLELPYTFGGGTKLEIKR-
|||||||||||||||||||||||||||||||||
SRVEAEDVGVYYCAQNLELPYTFGGGTKLEIKRA
```

1RZ7 Heavy

```
EVQLVQSGAEVKKPGATVKISCKASGYTFSDFYMYWVRQAPGKGLEWMGLIDPEDADTMYAEKFRGRVTITADTSTDTGY
|||||||||||||||||||||||*|||||*|*||*||*||||||||||||*||||**|*|||||*|||||||||||||*|
EVQLVQSGAEVKKPGATVKISCKVSGYTFTDYYMHWVQQAPGKGLEWMGLVDPEDGETIYAEKFQGRVTITADTSTDTAY

LELSSLRSEDTAVYYCAADPWELNAFNVWGQGTLVSVSS
*||||||||||||||||*||||||*|**|||||||*| |
MELSSLRSEDTAVYYCATDPWELNYFDYWGQGTLVTV-S
```

1RZ7 Light

```
IQMTQSPSSVSASVGDRVTITCRASQDISTWLAWYQQKPGKAPKLLIYAASTLQSGVPSRFSGSGSGTDFSLTINSLQPE
||||||||||||||||||||||||||*||*|||||||||||||||||||||*||||||||||||||||||*|||*|||||
IQMTQSPSSVSASVGDRVTITCRASQGISSWLAWYQQKPGKAPKLLIYAASSLQSGVPSRFSGSGSGTDFTLTISSLQPE

DFATYYCQQANSF-FTFGGGTKVEIKRT
||||||||||||| *|||||||||||||
DFATYYCQQANSFPLTFGGGTKVEIKRT
```

1T2Q Heavy

```
EVQLLEESGPGLVQPSQSLSITCTVSGFSLTSYGVHWVRQSPGKGLEWLGVIWSGGSTDYNAAFISRLSISKDNSKSQVF
|***|**|||||||||||||||||||||||||||||||||||||||||||||||||||||||||||||||||||||||||
EQVQLKQSGPGLVQPSQSLSITCTVSGFSLTSYGVHWVRQSPGKGLEWLGVIWSGGSTDYNAAFISRLSISKDNSKSQVF

FKMNSLQADDTAIYYCARNRGYSYAMDSWGQGTSVTVS
||||||||||||||||||||||*||||*||||||||||
FKMNSLQADDTAIYYCARNRGYYYAMDYWGQGTSVTVS
```

1T2Q Light

```
ELVMTQSPLSLPVSLGDQASISCRSSQSLVHSSGNTYLHWYLQKPGQSPKLLIYKVSNRFSGVPDRFSGSGSGTDFTLTI
***|||*|||||||||||||||||||||*|||*|||||*|||||||||||||||||||||||||||||||||||||||*|
DVLMTQTPLSLPVSLGDQASISCRSSQSIVHSNGNTYLEWYLQKPGQSPKLLIYKVSNRFSGVPDRFSGSGSGTDFTLKI

SRVEAEDLGVYYCFQGSHVPLTFGAGTKLELKR-
|||||||||||||||||||||||||||||||||
SRVEAEDLGVYYCFQGSHVPLTFGAGTKLELKRA
```

2AGJ Heavy

```
VTLKESGPTLVKPTQTLTLTCTFSGFSLTTTGEGVGWIRQPPGKALEFLAFIYWNDAKRYNPSLQSRLTITKDASKKQVVL
*|||||||||||||||||||||||||||*|*|*||||||||||||||*||*|||||*|||*|||*||||||||*||*||||
ITLKESGPTLVKPTQTLTLTCTFSGFSLSTSGVGVGWIRQPPGKALEWLALIYWNDDKRYSPSLKSRLTITKDTSKNQVVL

TLTNLDPVDTATYYCARTSGWDIEFEYWGQGTLVTVS
|*||*|||||||||||**|||||*|*|||||||||||
TMTNMDPVDTATYYCAHRSGWDIYFDYWGQGTLVTVS
```

2AGJ Light

```
EIVLTQSPGTLSLSPGERATLSCRASETVSNDKVAWYQQKPGQAPRLLIYGASSRATGIPDRFSGSGSGTDFTLSISGLEPE
||||||||||||||||||||||||||**||****||||||||||||||||||||||||||||||||||||||||*||*||||
EIVLTQSPGTLSLSPGERATLSCRASQSVSSSYLAWYQQKPGQAPRLLIYGASSRATGIPDRFSGSGSGTDFTLTISRLEPE

DFVVYYCQQYASSPRTFGQGTKVEIKRT
||*|||||||*|||*||||||||||||*
DFAVYYCQQYGSSPWTFGQGTKVEIKRL
```

3KYM Heavy

```
EVQLLESGGGLVQPGGSLRLSCAASGFTFSIYPMFWVRQAPGKGLEWVSWIGPSGGITKYADSVKGRFTISRDNSKNTLYLQ
||||||||||||||||||||||||||||||*|*|*||||||||||||||*|**|||*|*|||||||||||||||||||||||
EVQLLESGGGLVQPGGSLRLSCAASGFTFSSYAMSWVRQAPGKGLEWVSAISGSGGSTYYADSVKGRFTISRDNSKNTLYLQ

MNSLRAEDTATYYCAREGHNDWYFDLWGRGTLVTVS
||||||||||*||||*||||*|||||||||||||||
MNSLRAEDTAVYYCAKEGHNYWYFDLWGRGTLVTVS
```

3KYM Light

```
DIQMTQSPGTLSLSPGERATLSCRASQSVSSYLAWYQQKPGQAPRLLIYDASNRATGIPARFSGSGSGTEFTLTISSLQSED
*|*|||||*|||*||||||||||||||||||*|||||||||||||||||*||*|||||||||||||||||||||||||||||
EIVMTQSPATLSVSPGERATLSCRASQSVSSNLAWYQQKPGQAPRLLIYGASTRATGIPARFSGSGSGTEFTLTISSLQSED

FAVYYCQQYDKWPLTFGGGTKVEIK
|||||||||**||||||||||||||
FAVYYCQQYNNWPLTFGGGTKVEIK
```

3QRG Heavy

```
ITLKESGPTLVKPTQTLTLTCTFSGFSLSTSGMGVSWIRQPPGKALEWLAHIYWDDDKRYNPSLKSRLTITKDTSKNQVVL
||||||||||||||||||||||||||||||||*||*||||||||||||||*|||||||||*||||||||||||||||||||
ITLKESGPTLVKPTQTLTLTCTFSGFSLSTSGVGVGWIRQPPGKALEWLALIYWDDDKRYSPSLKSRLTITKDTSKNQVVL

TMTNMDPVDTATYYCARLYGFTYGFAYWGQGTLVTVS
||||||||||||||||**|||||*|*|||||||||||
TMTNMDPVDTATYYCAHRYGFTYYFDYWGQGTLVTVS
```

3QRG Light

```
DIVMTQSPDSLAVSLGERATINCRASQSVDY--NGISYMHWYQQKPGQPPKLLIYAASNPESGVPDRFSGSGSGTDFTLTI
|||||||||||||||||||||||**||||*|  |***|**|||||||||||||||*||**|||||||||||||||||||||
DIVMTQSPDSLAVSLGERATINCKSSQSVLYSSNNKNYLAWYQQKPGQPPKLLIYWASTRESGVPDRFSGSGSGTDFTLTI

SSLQAEDVAVYYCQQIIEDPWTFGQGTKVEIKR-
|||||||||||||||****||||||||||||||
SSLQAEDVAVYYCQQYYSTPWTFGQGTKVEIKRT
```

4YGV Heavy

```
QVQLVQSGAEVKKPGASVKVSCKASGYTFTDYYMHWVRQAPGQGLEWMGETNPRNGGTTYNEKFKGKATMTRDTSTSTAYM
||||||||||||||||||||||||||||||*||||||||||||||||||**||**|*|*|**||*|**||||||||||*||
QVQLVQSGAEVKKPGASVKVSCKASGYTFTSYYMHWVRQAPGQGLEWMGIINPSGGSTSYAQKFQGRVTMTRDTSTSTVYM

ELSSLRSEDTAVYYCTIGTSGYDYFDYWGQGTLVTVS
|||||||||||||||**||||||||||||||||||||
ELSSLRSEDTAVYYCARGTSGYDYFDYWGQGTLVTVS
```

4YGV Light

```
DIVMTQTPLSLSVTPGQPASISCRSSQSIVHSDGNIYLEWYLQKPGQSPKLLIYKVSYRFSGVPDRFSGSGSGTDFTLKIS
|||||||||||||||||||||||*||||**||||**||*||||||||||*||||*||*|||||||||||||||||||||||
DIVMTQTPLSLSVTPGQPASISCKSSQSLLHSDGKTYLYWYLQKPGQSPQLLIYEVSSRFSGVPDRFSGSGSGTDFTLKIS

RVEAEDVGVYYCFQASHVPYTFGQGTKLEIKR-
||||||||||||*|**|*||||||||||||||
RVEAEDVGVYYCMQGIHLPYTFGQGTKLEIKRT
```

1.3 Comparison of Flexibility Calculations Across Ensemble Generation Methods

In this work, we have considered multiple ensemble generation methods in conjunction with FIRST-PG analysis to determine the flexibility of CDR-H3 loops. Of all methods used in this paper to generate structural ensembles, only MD simulations have previously been coupled with flexibility analysis[8–11]. Rosetta FastRelax, KIC, and RosettaAntibody have not been used previously for flexibility analysis. MD simulations permit for fluctuations between low-energy states and variations in hydrogen bonding networks, effectively capturing the "flickering" nature of hydrogen bonds. The Rosetta-based methods consider hydrogen-bonding energy, but do not involve dynamic motion in the same way as MD simulations. The Rosetta FastRelax protocol generates ensembles representative of the local energy minimum through side-chain repacking and gradient-based energy minimization, so flexibility analysis of these ensembles should be comparable to flexibility analysis of crystal structures. Rosetta KIC on the other hand generates ensembles of low-energy CDR-H3 loop conformations by *de novo* modeling of the CDR-H3 loop. RosettaAntibody generates ensembles of low-energy antibody conformations, building on KIC motions through additional V_H–V_L docking. We compared FIRST-PG calculations on Rosetta FastRelax, RosettaAntibody, and MD ensembles, for three well-studied antibodies, excluding KIC ensembles from analysis because they are effectively superseded by RosettaAntibody ensembles. Qualitatively, the FIRST-PG results agree for all methods for 48G7 and the anti-fluorescein antibody. The Rosetta FastRelax results differ from the other methods for the anti-influenza, with the naïve showing significantly more rigidity. This is most likely due to the difference in quality between the crystal structures. Quantitatively, the ΔAUC values for RosettaAntibody and MD ensembles for all three antibody pairs compare well (Supplemental Table 2). Additionally, we compared only RosettaAntibody and MD ensembles for three naïve-reverted/mature antibody pairs, where we found that the ΔAUC values roughly agree for two out of three antibody pairs. Taken together, these results indicate that flexibility analyses on RosettaAntibody homology model ensembles are similar to analyses on MD ensembles.

2 Supplementary Figures and Tables

2.1 Supplementary Figures

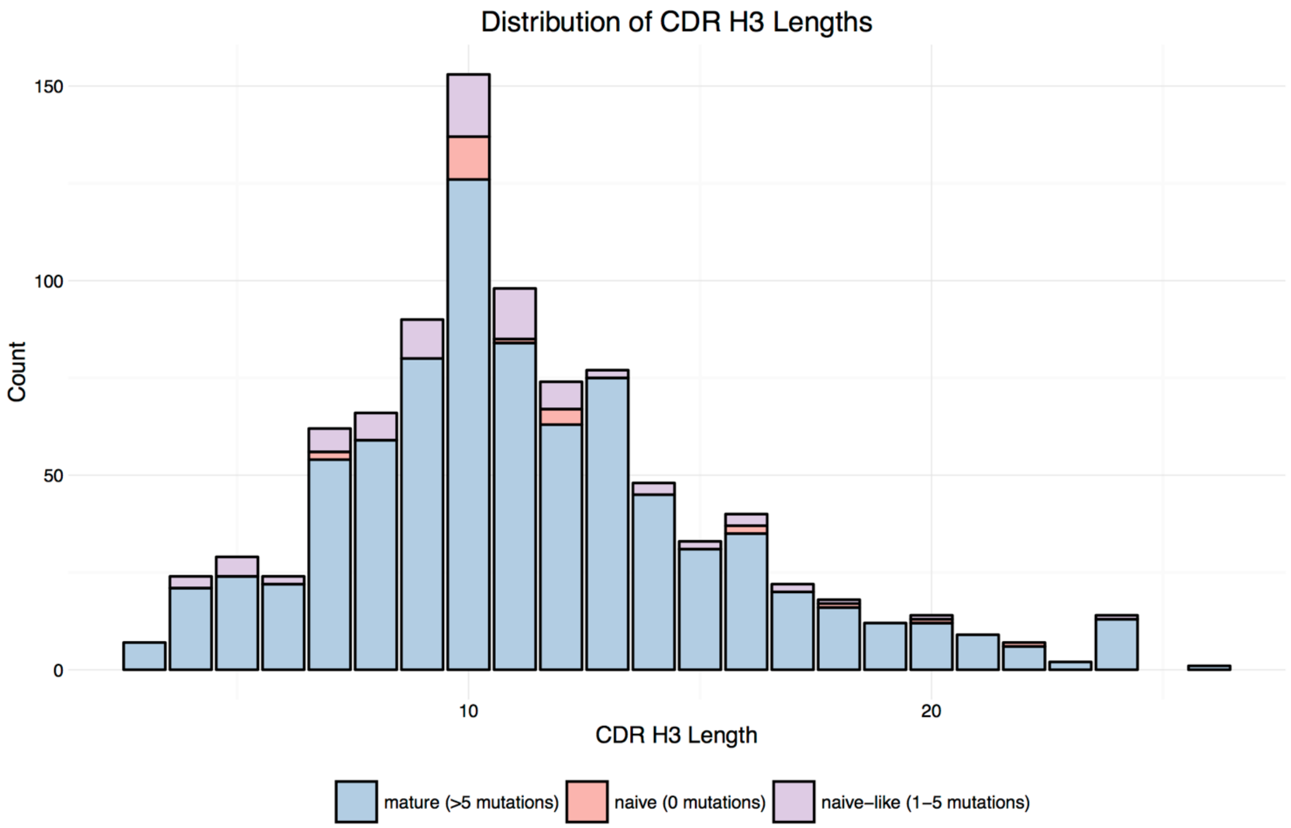

Supplementary Figure 1. Counts of CDR-H3 loop lengths in our crystallographic data set. Colors indicate the number of mutations. The most common loop length is ten, while there is a wide range of loop lengths in the crystallographic data set.

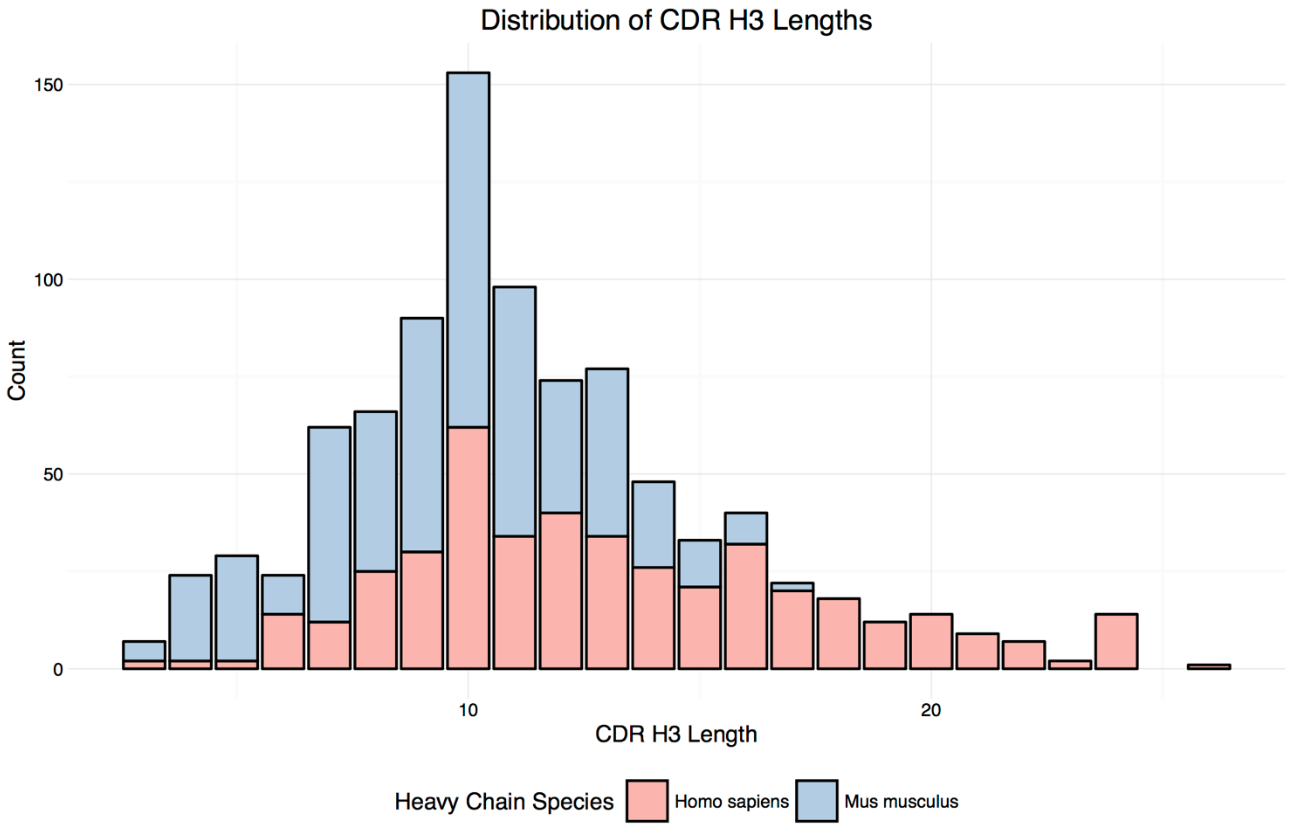

Supplementary Figure 2. Counts of CDR-H3 loop lengths in our crystallographic data set. Colors indicate the species from which the antibody heavy chain is derived. As previously observed, we see that human antibodies have, on average, longer CDR-H3 loops than mouse antibodies.

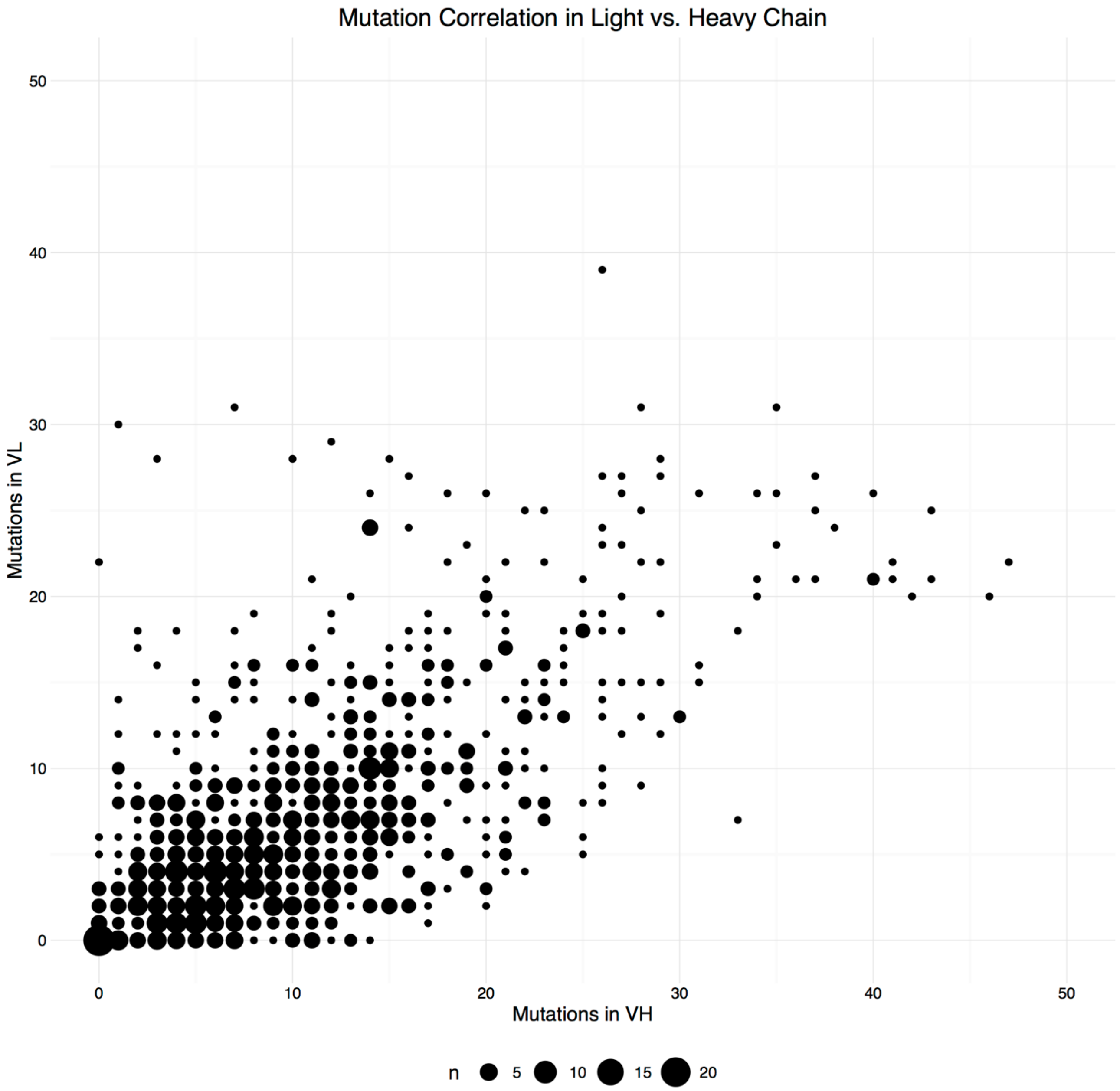

Supplementary Figure 3. Distribution of mutations in our crystallographic data set. The size of the point represents the number of antibodies possessing that count of heavy and light chain mutations. As expected, mutations are more frequent in the heavy chain than in the light chain.

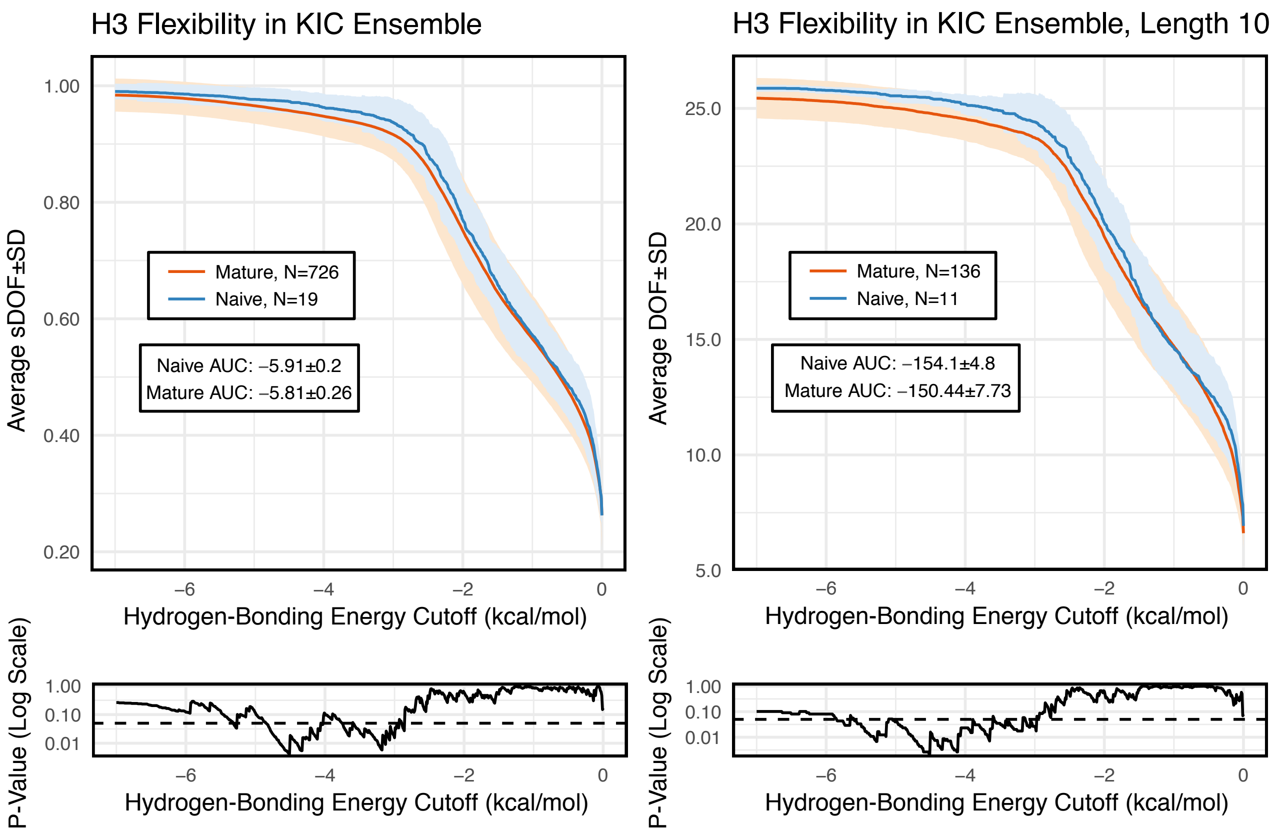

Supplementary Figure 4. FIRST-PG analysis of KIC ensembles of the crystallographic antibody set, with naïve antibody data shown in blue and mature antibody data shown in orange and standard error of the mean shown in a lighter shade of the respective color. Subplots, below each main plot, show the p-value computed by a KS comparison of the naïve and mature DOF distributions for each hydrogen-bonding energy cutoff, with null hypothesis being that the distributions are the same and a dashed line indicating a p-value of 0.05. (Left) When comparing DOFs scaled to a theoretical maximum as a function of hydrogen-bonding energy cutoff for the entire set, the values are similar for both naïve (AUC = –5.9 ± 0.2) and mature (AUC = –5.8 ± 0.3) antibodies. (Right) Comparison of DOFs for a single length without scaling reveals naïve antibodies to possess a slightly higher DOF value than mature antibodies at the same hydrogen-bonding energy cutoff. AUCs however are within a standard deviation, compare naïve at −154.1 ± 4.8 and mature at −150.4 ± 7.7.

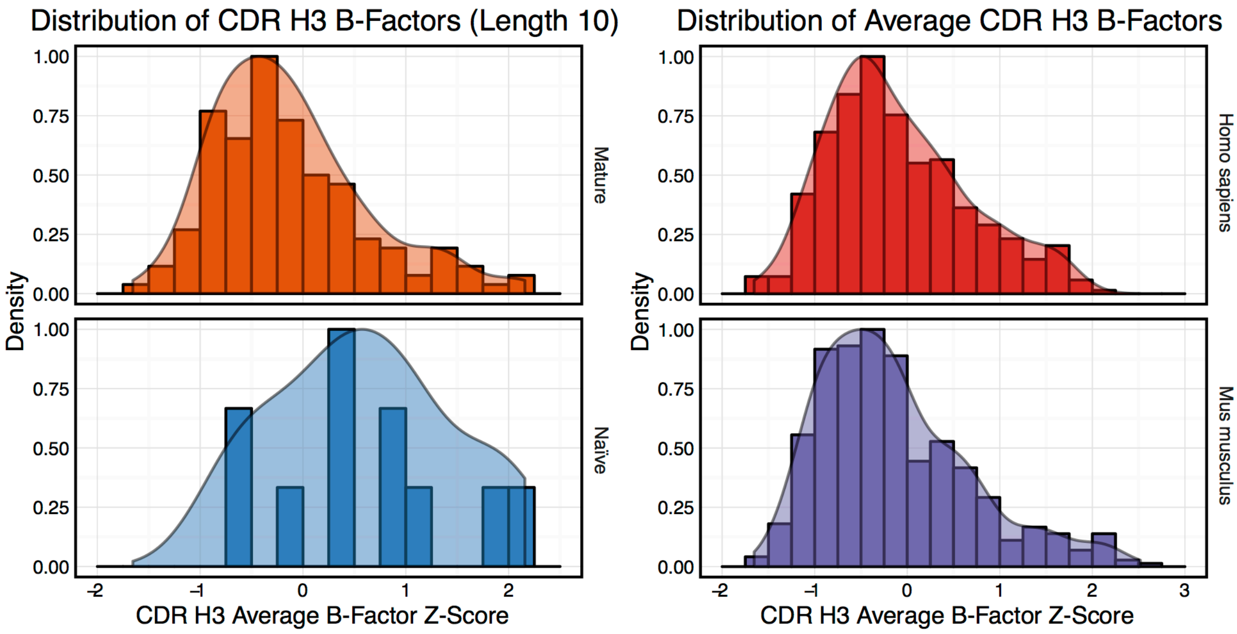

Supplementary Figure 5. Average CDR-H3 loop B-factor z-score for antibodies with loops of length 10 split by number of mutations (left) and all antibodies split by heavy-chain species (right). Mature antibodies have at least one mutation. Comparing the difference in mature versus naïve means for length 10 CDR-H3 loops only to a randomized test (as described in the methods) shows only ~2.9% of random permutations have an equal or greater difference. A two-sample KS test yields a p-value of 0.0135 and D of 0.4949, so these distributions appear to be on the threshold of significance. However, that is obviated when bound structures are excluded from analysis, resulting in ~4.8% of random permutations having an equal or greater difference in means than the observed and a KS-test p-value of 0.0989 (with D of 0.3989). It is difficult to quantify if there is a difference in the length 10 set due to low counts (only 11 naïve antibodies), whereas there is no visible difference between the human and mouse antibodies (21.3% of random permutations have an equal or greater difference and the KS-test p-value is 0.6654 [with a D of 0.0748]).

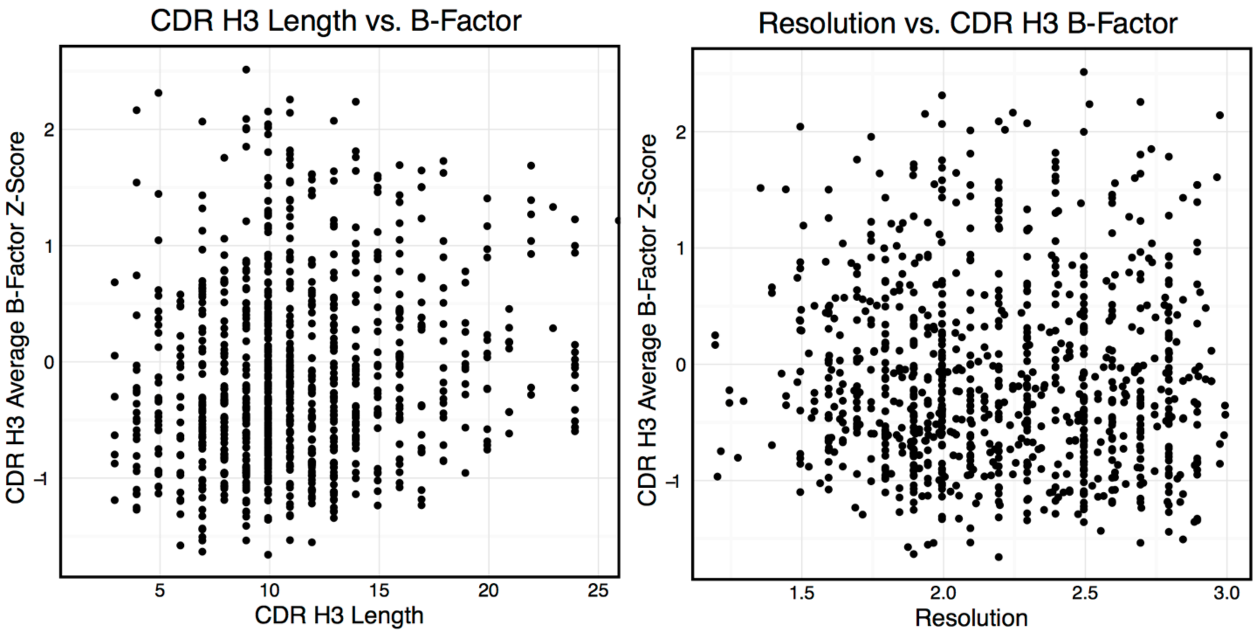

Supplementary Figure 6. Average CDR-H3 loop B-factor z-score compared with either loop length (left) or crystal structure resolution (right). There is not an obvious dependence of CDR-H3 loop B-factor z-score on either.

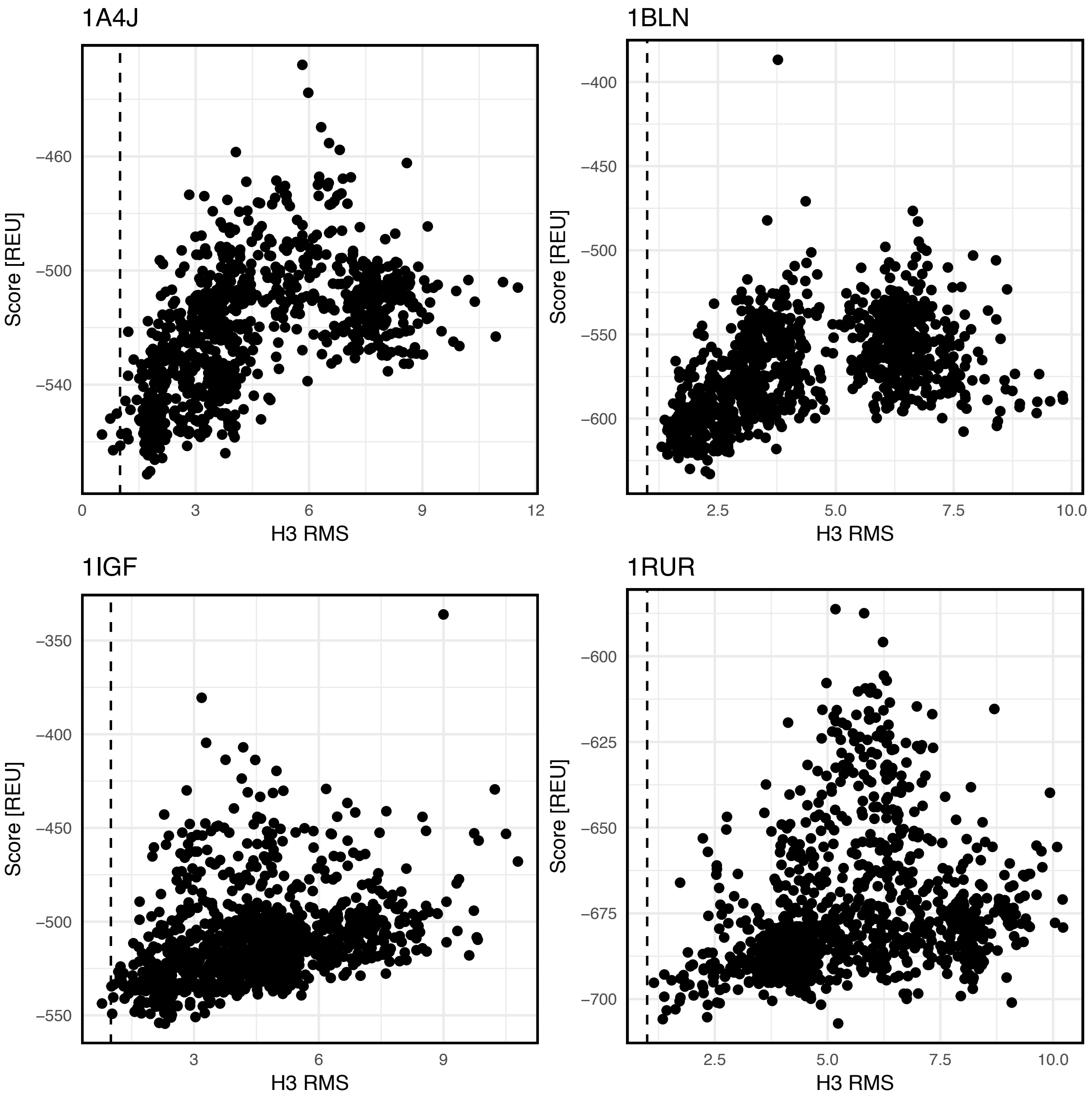

Supplementary Figure 7. Accuracy of CDR-H3 loop modeling for the paired mature–reverted-naïve antibodies. The loop RMSD versus model energy is shown in what is known as a "funnel plot" for four mature antibodies for which structures are known. Dashed line indicates 1 Å RMSD. Model quality is good for 1A4J and 1IGF with multiple sub-angstrom CDR-H3 loop models at relatively low-energies, while model quality is ok for 1BLN and 1RUR with multiple low-energy models close to achieving sub-angstrom accuracy.

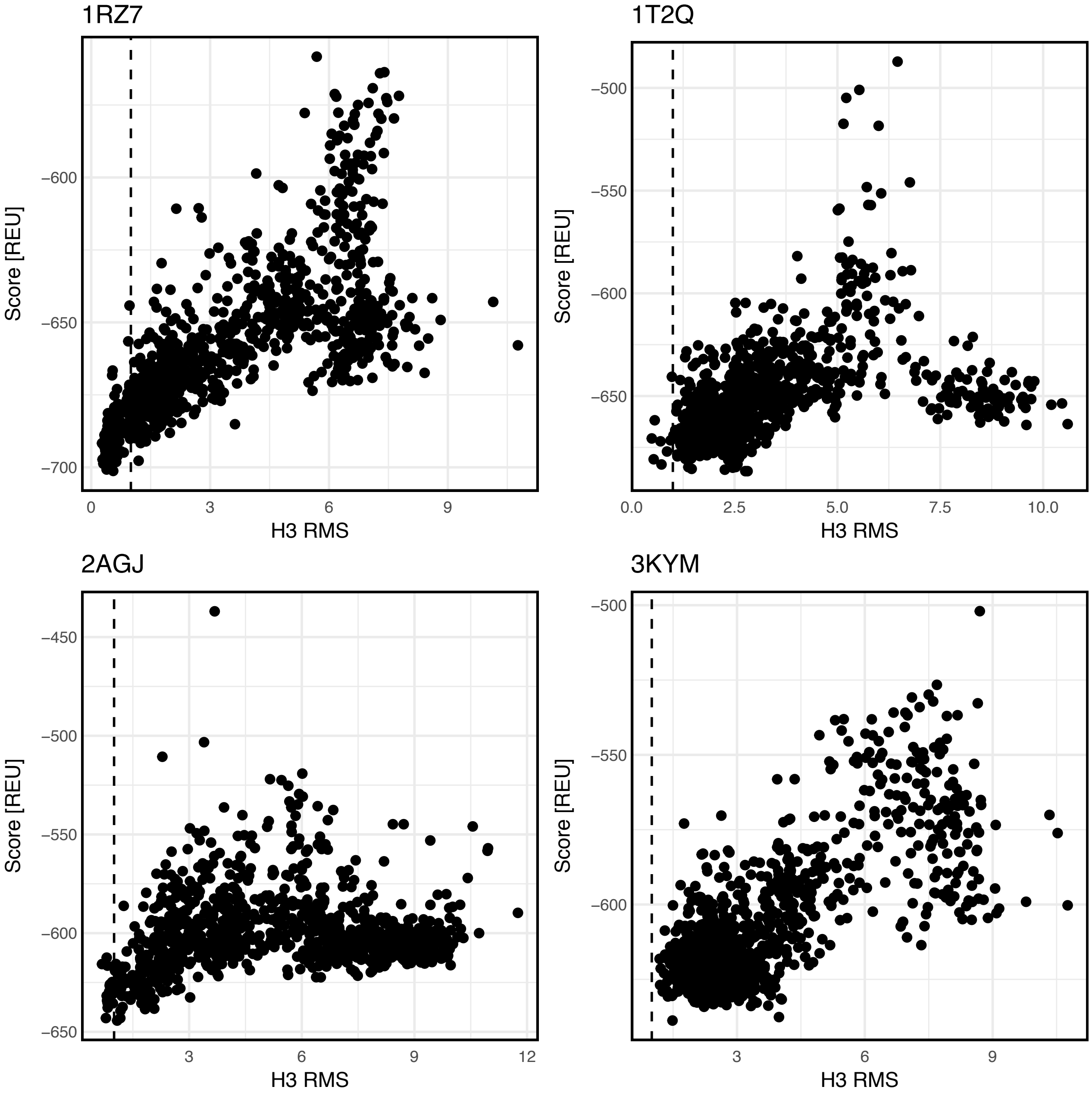

Supplementary Figure 8. As in Supplementary Figure 7, accuracy of CDR-H3 loop modeling for the paired mature–reverted-naïve antibodies. The loop RMSD versus model energy is shown in what is known as a “funnel plot” for four mature antibodies for which structures are known. Dashed line indicates 1 Å RSMD, which is an excellent result for *de novo* loop modeling. Model quality is good for all but 3KYM, with multiple sub-angstrom CDR-H3 loop models at relatively low-energies, while model quality is ok for 3KYM with multiple low-energy models close to achieving sub-angstrom accuracy.

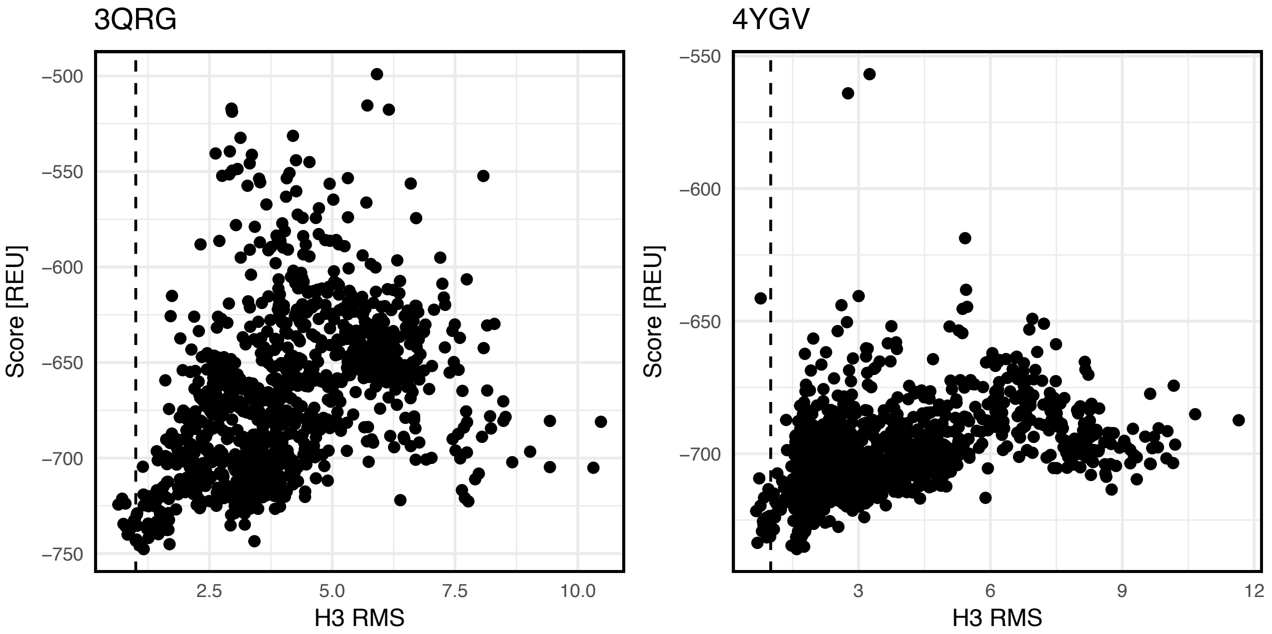

Supplementary Figure 9. As in Supplementary Figures 7 and 8, accuracy of CDR-H3 loop modeling for the paired mature–reverted-naïve antibodies. The loop RMSD versus model energy is shown in what is known as a "funnel plot" for two mature antibodies for which structures are known. Dashed line indicates 1 Å RSMD, which is an excellent result for *de novo* loop modeling. Model quality is good for both 3QRG and 4YGV, with multiple sub-angstrom CDR-H3 loop models at relatively low-energies.

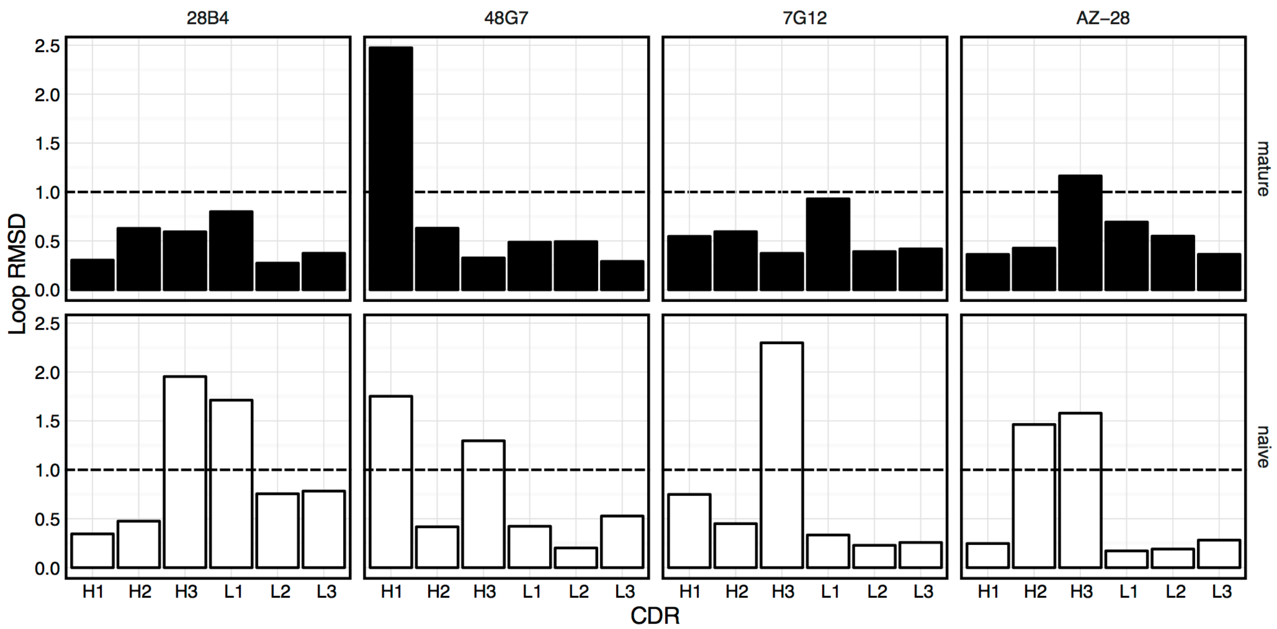

Supplementary Figure 10. CDR loop motions upon antigen binding for four catalytic antibodies. Loop RMSDs (in angstroms) were calculated from the difference in Cα atom positions after alignment of the corresponding (heavy or light) framework Cα atoms. The dashed line indicates 1 Å. PDB IDs for the structures used in these calculations are reported in Supplementary Table 3. For naïve antibodies, greater than 1 Å motions frequently occur in the CDR-H3 loop for all four antibodies, whereas similar motion rarely occurs for the mature equivalents.

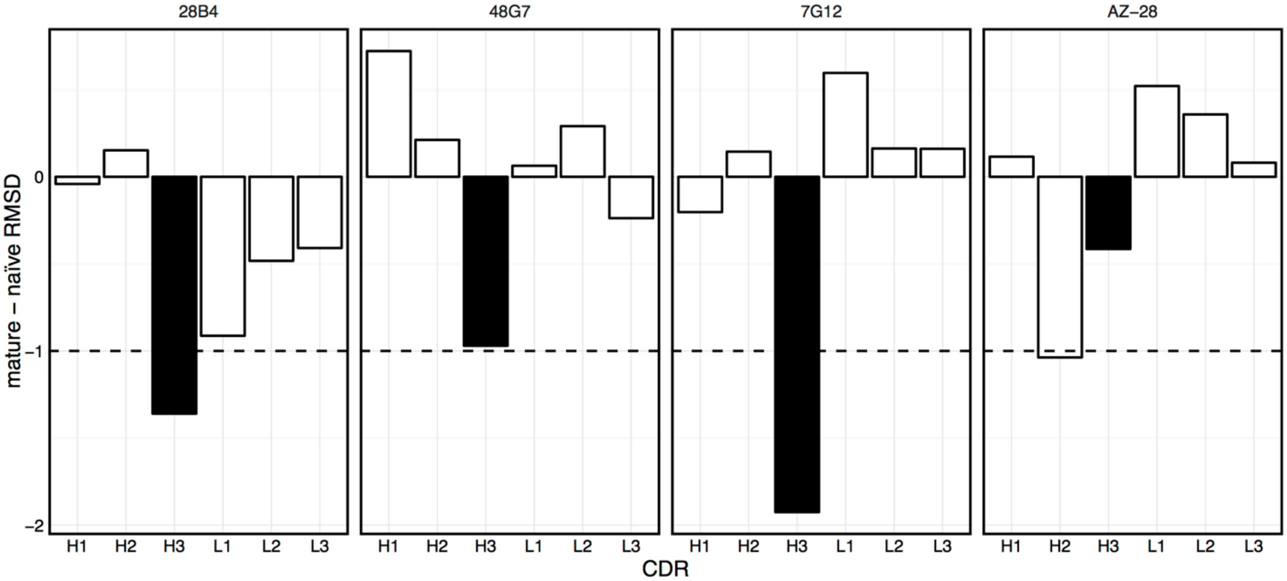

Supplementary Figure 11. Difference in CDR loop motions upon antigen binding between naïve and mature antibodies for four catalytic antibodies. RMSD calculations were done in the same manner as for Supplementary Figure 1. The CDR-H3 loops is highlighted in black. The dashed line indicates 1 Å. A more negative value here indicates less motion upon binding in the mature antibody. The effects of affinity maturation on CDR-H3 loop motion in crystal structures are not always significant, with only 2/4 showing motion reduction greater than an angstrom.

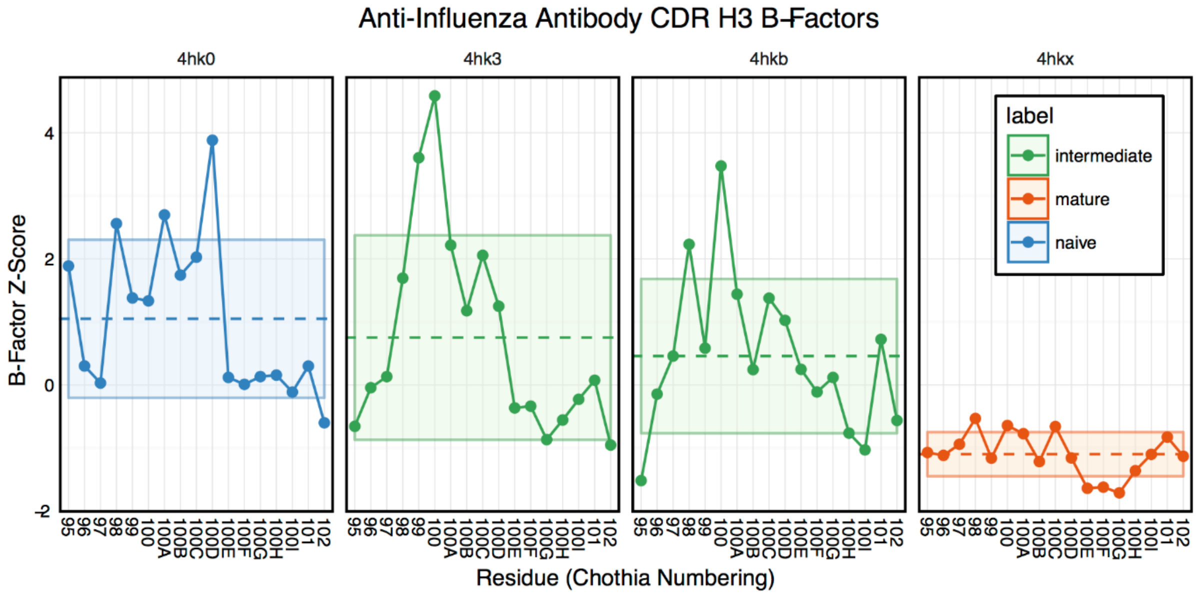
Anti-Influenza Antibody CDR H3 B-Factors
4hk0
4hk3
4hkb
4hkx
label
intermediate
mature
naive
B-Factor Z-Score
Residue (Chothia Numbering)

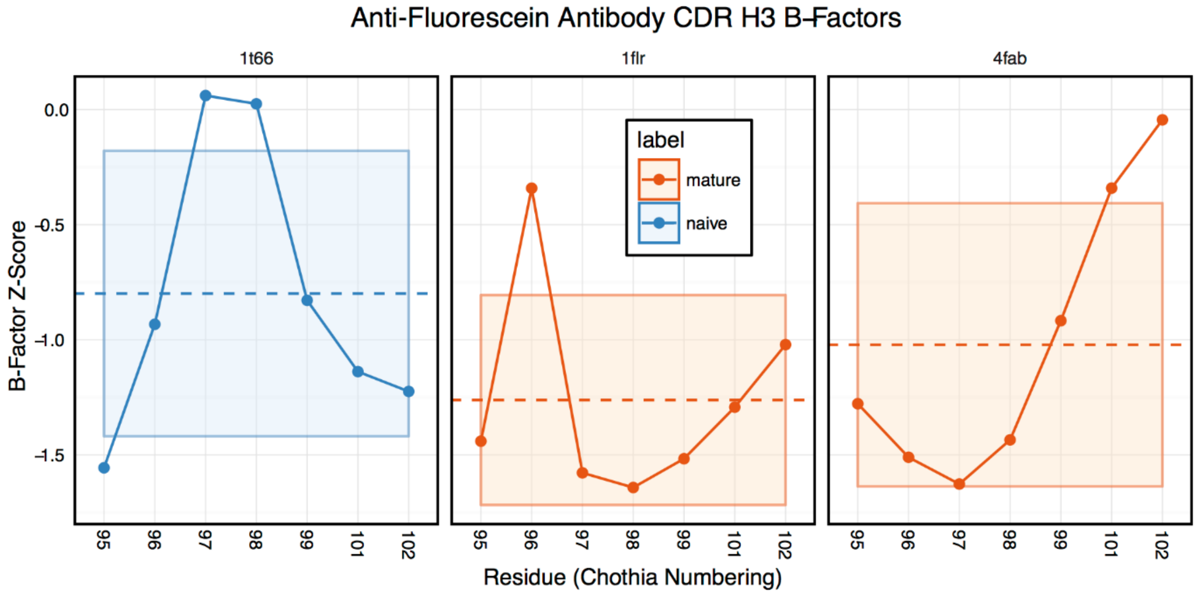
Anti-Fluorescein Antibody CDR H3 B-Factors
1t66
1flr
4fab
label
mature
naive
B-Factor Z-Score
Residue (Chothia Numbering)

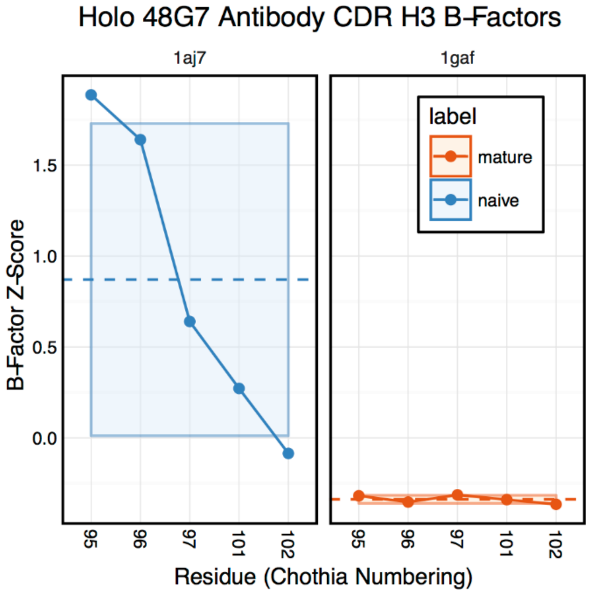
Holo 48G7 Antibody CDR H3 B-Factors
1aj7
1gaf
label
mature
naive
B-Factor Z-Score
Residue (Chothia Numbering)

Supplementary Figure 12. (Previous page.) CDR-H3 loop B-factor z-scores for three previously studied antibodies, with PDB IDs shown above each plot. B-factor z-scores were calculated with respect to the F_v region and for Cα atoms only. The anti-influenza antibodies have vary in resolution from 2.5 Å for the naïve and mature to 3.0 Å (4HK3) and 3.6 Å (4HKB) for the intermediates. Additionally, the mature anti-influenza antibody has antigen bound affecting the CDR-H3 loop B-factors. We can see that affinity maturation does not always lead to a reduction in CDR-H3 loop B-factor z-scores.

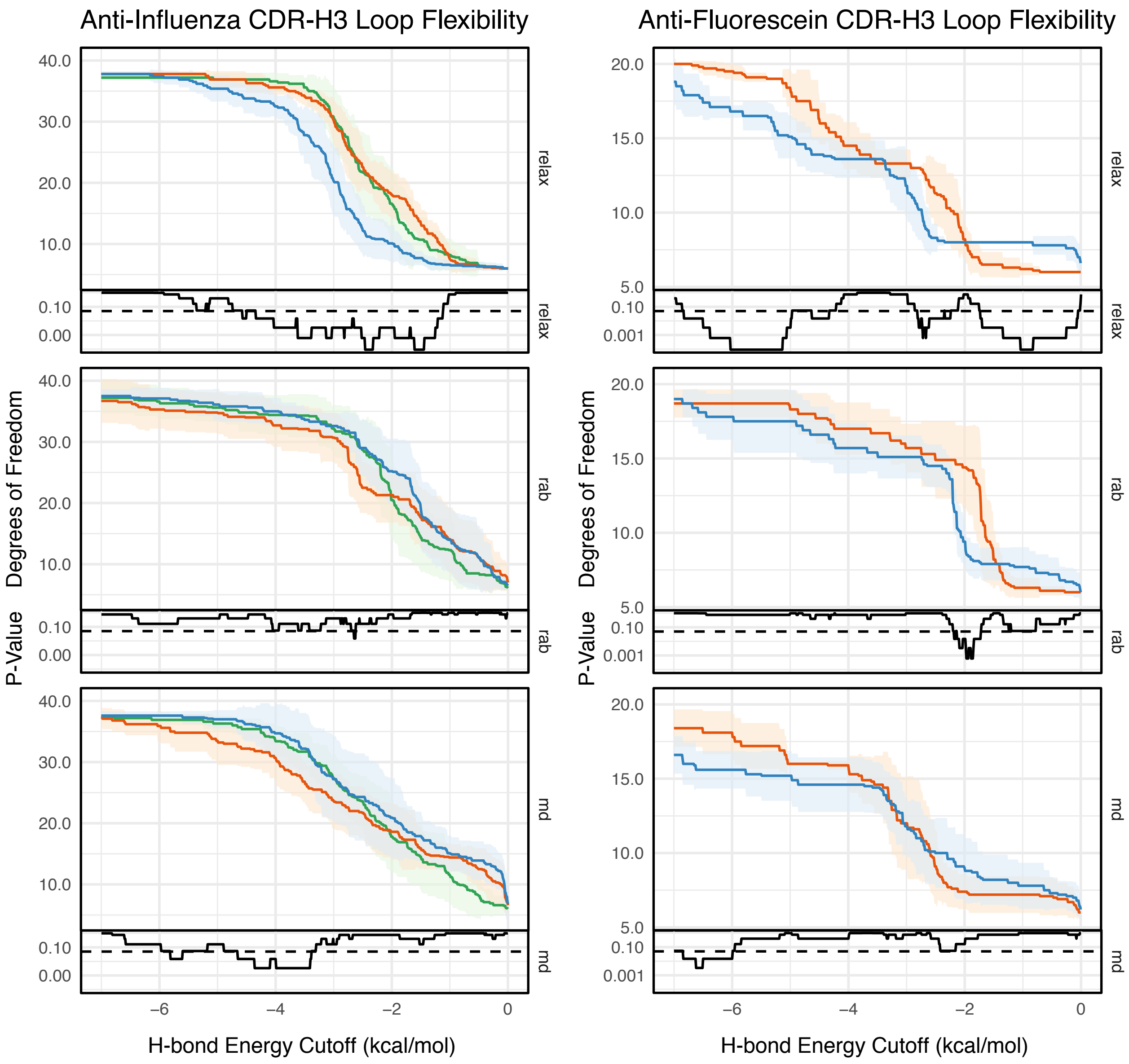

Supplementary Figure 13. FIRST-PG analysis of two previously studied antibodies with MD simulations (labelled MD), RosettaAntibody (labelled RAB), and Rosetta FastRelax (labelled relax) used to generate structural ensembles. Naïve antibodies are colored blue and mature antibodies are colored red, while an "intermediate" (4HKB) influenza antibody is shown in green. Subplots, below each main plot, show the p-value computed by a KS comparison of the naïve and mature DOF distributions for each hydrogen-bonding energy cutoff, with null hypothesis being that the distributions are the same and a dashed line indicating a p-value of 0.05. Again, the effects of affinity maturation are not obvious.

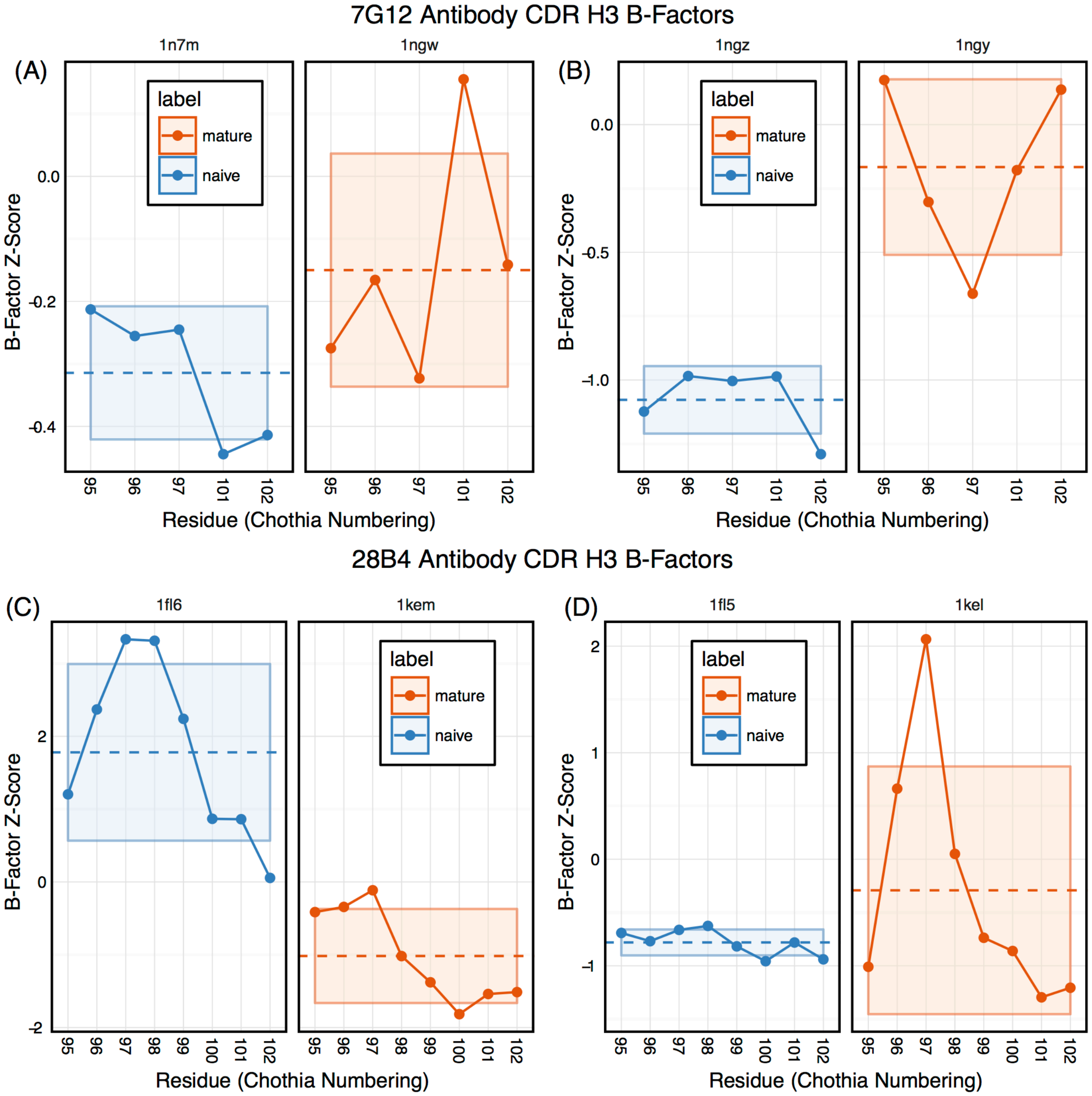

Supplementary Figure 14. CDR-H3 B-factor z-scores for antigen-bound and free crystal structures of catalytic antibodies 7G12 and 28B4. The 7G12 antibody has higher z-scores for the mature than the naïve antibody for both the (A) unbound and (B) bound structures, indicating a gain in flexibility upon maturation. The 28B4 antibody shows a loss of flexibility upon maturation for the unbound structure comparison (C), but no change in the bound structure comparison (D).

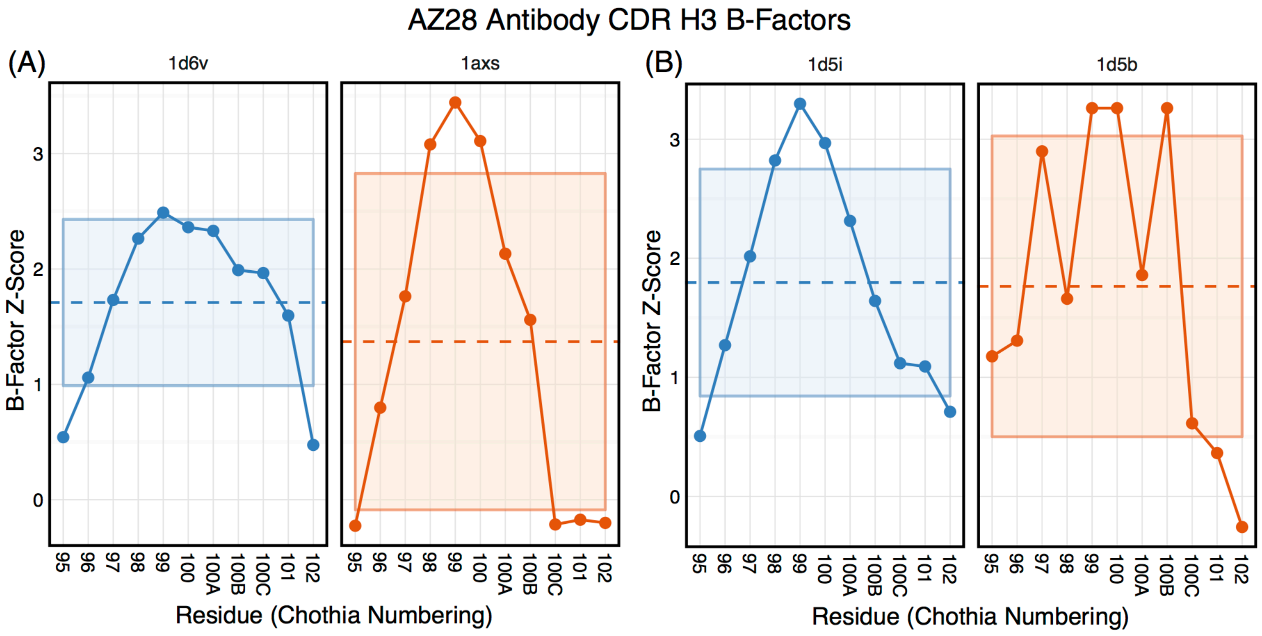

Supplementary Figure 15. CDR-H3 loop B-factor z-scores for antigen-bound and free crystal structures of the catalytic antibody AZ-28 reveal no significant difference between the naïve and mature antibodies.

2.2 Supplementary Tables

Supplementary Table 1. List of antibodies analyzed in this study. The following 922 antibodies were studied (attached separately).

Supplementary Table 2. Rigidity changes according to several methods. Changes in the rigidity of the 48G7 antibody CDR-H3 loop according to several methods. Unbound is denoted by (U) and bound is denoted by (B). A positive number indicates an increase in rigidity upon affinity maturation. Changes for B-factors are calculated as the difference in the average CDR-H3 loop B-factor between the naïve and mature crystal structure: $\Delta B = \overline{B_{\text{naive}}} - \overline{B_{\text{mature}}} \pm \sqrt{s_{\text{naive}}^2 + s_{\text{mature}}^2}$. Changes in FIRST-PG are calculated as the percent change between the AUC of the CDR-H3 melting curve for naïve and mature antibodies: $\Delta AUC = 100 \times \frac{\text{AUC}_{\text{mature}} - \text{AUC}_{\text{naive}}}{\text{AUC}_{\text{naive}}}$. Finally, changes in MD RMSD or RMSF are calculated as the difference in average CDR-H3 loop RMSF or RMSD between the MD simulations of the naïve and mature antibodies: $\Delta R = \overline{R_{\text{naive}}} - \overline{R_{\text{mature}}} \pm \sqrt{s_{\text{naive}}^2 + s_{\text{mature}}^2}$. (*) Only bound crystal structures were available for the 4-4-20 antibody, but Relax, KIC, RA and MD simulations were run without antigen. (#) Only an unbound naïve and bound mature crystal structures were available for the anti-influenza antibody, but Relax, KIC, RA and MD simulations were run without antigen.

Antibody	ΔB-Factor	ΔRelax AUC (%)	ΔKIC AUC (%)	ΔRA AUC (%)	ΔMD RMSD	ΔMD RMSF	ΔMD AUC (%)
48G7 (U)	2.14 ± 0.62	3.9	13.0	0.2	−1.04 ± 0.65	−0.71 ± 0.64	−1.3
48G7 (B)	1.21 ± 0.89	−6.2	−8.9	-	-		-
4-4-20 (U)	-	-	-	−6.2	0.85 ± 0.53	−0.35 ± 0.36	−4.1
4-4-20 (B)*	0.46 ± 0.77	−8.4	2.8	-			-
Influenza (U)	-	-	-	6.1	2.25 ± 1.33	0.44 ± 1.14	9.1
Influenza (B)#	1.85 ± 1.64	−15.2	1.7	-			-

Supplemental Table 3. Incomplete list of previously studied naïve–mature antibody pairs, with crystal structures. The naïve antibody here does not always have zero mutations, but rather was designated as naïve by the authors of the original study. Crystal structures may have unresolved residues. Asterisk (*) indicates a catalytic antibody. Additional crystal structures, not listed in the table, include: [1]1Q9L and 1Q9V; [2]1Q9R and 1Q9T; [3]1Q9T; [4]1FLR; [5]4HK3 (unbound intermediate); [6]4FQ2; and [7]4S1R and 4S1S. Antibody 4JPK[8] is bound to a designed antigen, rather than the natural one.

		Naïve		Mature		
Antibody	H3 Length	Unbound	Bound	Unbound	Bound	References
7G12*	5	1NGZ	1N7M	1NGY	1NGW	Yin et al.[12]
28B4*	8	1FL5	1FL6	1KEL	1KEM	Hsieh-Wilson et al.[13] and Yin et al.[14]
AZ-28*	11	1D5I	1D6V	1D5B	1AXS	Ulrich et al.[15] and Mundorff et al.[16]
S25-2/S45-18	11	1Q9K[1]	1Q9Q[2]	1Q9O	1Q9W[3]	Nguyen et al.[17]
48G7*	5	2RCS	1AJ7	1HKL	1GAF	Wedemayer et al.[18,19] and Patten et al.[20]
D44.1/F10.6.6	7	1MLB	1MLC	2Q76	1P2C	Braden et al.[21], Acierno et al.[22], and Cauerhff et al.[23]
H26/H63/H8	5	1DQQ	1DQJ/1NDM	-	1NDG	Li et al.[24,25]
4-4-20	7	-	1T66	-	4FAB[4]	Terzyan et al.[26] and Herron et al.[27]
Anti-influenza	17	4HK0	-	4HKB[5]	4HKX	Schmidt et al.[28]
PGT121	24	4FQQ	-	4FQ1[6]	4FQC	Mouquet et al.[29]
NIH45-46	16	4JDV	4JDT	3U7W	3U7Y	Scharf et al.[30] and Diskin et al.[31]
VRC01	12	4JPI	4JPK[8]	-	4S1Q[7]	Jardine et al.[32] and Wu et al.[33]
VRC03	14	5JOF	-	5JXA	3SE8	Wu et al.[34] and Davenport et al.[35]
VRC26	36	4ODH	-	4OD1	-	Doria-Rose et al.[36]

Supplementary Table 4. Brief summaries of previous work considering the effects of affinity maturation on antibody flexibility.

	Authors	Year	Journal	Short Summary
0	Foote and Milstein	1994	PNAS	Stopped-flow fluorescence measurements on three antibodies reveal binding kinetics with multiple phases indicating that "ligand binding involved isomerization, as well as associative steps." The experimentally characterized antibodies were mature, but the authors speculate that "antibodies in the primary repertoire may be more prone to isomerism" and "affinity maturation in such cases may include mutaitons leading to a more favorable isomeric equilibrium."[20]
1	Patten et al.	1996	Science	To our knowledge, the first published suggestion of rigidification of the CDR H3 upon maturation, based on studies of the esterolytic antibody **48G7**, "affinity maturation appears to play a conformational role, either in reorganizing the active site geometry or limiting side-chain and backbone flexibility of the germline antibody." But no direct evidence is presented.[18]
2	Wedemayer et al.	1997	Science	This paper reports crystal structures for hapten bound/unbound and naïve/mature **48G7** antibodies. Comparison of naïve/mature structural rearrangements upon binding reveals reduced CDR H3 motion of the mature antibody. The authors conclude, "The end result of these somatic mutations is a combining site with improved complementarity to hapten … which, in contrast to the germline antibody, binds hapten in a pre-organized fashion."[37]
	Chong et al.	1999	PNAS	500 ps MD simulation on 48G7 antibody with hapten present found higher RMSFs in the "belly" atoms from the naïve than the mature antibody.[16]
3	Mundorff et al.	2000	Biochemistry	Similar to paper #2, this paper compares the naïve/mature bound/unbound structures of catalytic antibody **AZ-28**. The authors find large rearrangements of the naïve CDR H3 upon hapten binding.[38]
4	Manivel et al.	2000	Immunity	In this paper, surface plasmon resonance is used to study **anti-peptide antibodies**. Comparison of the naïve/mature enthalpic and entropic contributions to binding reveals that reduction of entropic contributions is the primary cause for increased affinity upon maturation. The authors conclude, "high affinity and specificity are simultaneously achieved by the simple device of regulating paratope flexibility."[14]
5	Yin et al.	2001	Biochemistry	Similar to papers #2 & #3, this paper compares naïve/mature bound/unbound structures of redox antibody **28B4** (half of which were solved in Hseih-Wilson et al. [PNAS, 1996]). The authors find that there is more motion in germline CDRs H3 and L1, than in the mature, concluding, "mutations introduced into the germline antibody … act to decrease the CDR loop flexibility and preorganize hapten

				binding."[39]
6	Jimenez et al.	2003	PNAS	Using a chromophore (fluorescein) and three-pulse photon echo peak shift (3PEPS) spectroscopy, this paper compares the dynamics of three antibody-fluorescein complexes (including **4-4-20**), demonstrating different mechanisms of antigen recognition, including lock-and-key, induced-fit, and conformational selection.[40]
7	Yin et al.	2003	JMB	Similar to papers #2, #3, & #5, crystal structures of naïve antibody **7G12** in complex with cognate hapten, non-cognate jeffamine, and ligand-free reveal that large changes in the CDR H3 loop permit binding of both molecules. Comparison to the mature antibody shows that the maturation process rigidifies the loop in the hapten-bound state and sterically occludes jeffamine binding.[24]
8	Li et al.	2003	NSM	To our knowledge, the first published study with protein antigen, rather than small molecules or peptides (as in #1–7). Authors compared crystal structures of **antibody–hen-egg-white-lysozyme** complexes for a high-affinity and low-affinity antibody. Additional structural comparison was made to crystal structures of unbound antibodies. SPR was used to determine binding parameters. Overall, the results show that binding is enhanced due to increased burial of apolar surface area and improved shape complementarity. Evidence of CDR H3 loop rearrangements is minimal.[41]
9	Jimenez et al.	2004	PNAS	Similar to #6, this paper uses 3PEPS to characterize the effects of mutations in the light chain on **4-4-20 antibody**–antigen complex flexibility. The authors find significant rigidification of the complex, as exhibited by decreased picosecond and nanosecond time-scale motions by 2.5- and 20-fold in the mature vs. naïve antibody, with the significant caveat that only the light chain was reverted.[42]
10	Zimmerman et al.	2006	PNAS	A follow-up paper on #9. Using 3PEPS, dynamic Stokes-shift (DSS), SPR, and MD experiments, this paper characterizes the evolution of the **antifluorescein antibody 4-4-20**. All experiments show that the naïve antibody exhibits more conformational heterogeneity than the mature antibody. MD shows that the naïve motion is primarily in the L1, L2, and H3 CDRs. Therefore, the conclusion is that evolution optimizes a binding site for a specific molecule by preconfiguration.[43]
11	Thorpe et al.	2007	PNAS	A follow-up paper on #10, with MD simulations of several (varying in maturation) bound/unbound **4-4-20 antibody**–antigen complexes. Analysis shows larger fluctuations in the germline unbound simulation than the bound. Calculations of the enthalpies and entropies show a larger change entropy (of binding) upon maturation than in enthalpy (of binding).[44]

12	Thielges et al.	2008	Biochemistry	This paper analyzes the thermodynamics and dynamics of six **antifluorescien antibodies** (including **4-4-20**). ITC is used to measure enthalpies and entropies of binding. 3PEPS and transient grading (TG) experiments are used to measure motions on the femto-, pico-, and nano-second timescales. Experiments show antibodies net favorable dG binding is due in some cases primarily to a favorable enthalpy change, but one case due to a favorable entropy change. The authors conclude that the immune system can generate antibodies with a variety of dynamics, from rigid antibodies capable of lock-and-key binding to flexible ones capable of induce-fit or conformational-selection binding.[45]
13	Babor et al.	2008	Proteins	In this paper, the authors use Rosetta Design on antibody crystal structures to recover CDR H3 sequences. When the design is constrained to multiple structures, then naïve sequence is more likely to be recovered than the mature sequence. This is taken to indicate that the naïve sequence is optimal for conformational flexibility.[46]
14	Wong et al.	2010	Proteins	In this paper, **four catalytic antibodies** and their naïve equivalents are modeled using MD simulations. Flexibility was assessed by comparing alpha-carbon B-factors for the six CDR loops. In three of the four studied antibodies, a loss of flexibility in residues contacting antigen was observed following maturation.[47]
15	Adhikary et al.	2012	JBC	This paper is similar to #12, in that a panel of antibodies was evolved against a chromophore (MPTS) and they were studied by ITC, 3PEPS, and TG. The results are similar to #12, in that the antibodies had varying dynamics and recognized MPTS through multiple mechanisms. The authors conclude that antibodies are initial dynamic with the potential to recognize multiple targets, but eventually are tailored to a single target by evolution.[28]
16	Schmidt et al.	2013	PNAS	This paper involves crystallization, MD simulation, and SPR studies of **broadly neutralizing influenza virus antibodies**. The authors show with ~30 microsecond MD simulations that the naïve antibody CDR H3 loop is rarely in the bound conformation, whereas the mature antibody CDR H3 loops occupy the bound conformation between 20–70% of the time. SPR shows that ~66% of the improvement in KD can be attributed to a 10-fold decrease in dissociation rate upon affinity maturation. The authors collectively interpret the results to indicate that "increased conformational restriction of the CDR H3 has been the principle consequence of affinity maturation."[48]
17	Willis et al.	2013	PLoS Comp. Bio.	Similar to #13, this paper uses multi/single-state design to assess the structural preference (sequence recovery) of naïve/mature sequences. The authors design on multiple structures originating from the same VH germline, diverging from the previous study. Their results indicate that multi-state design is more likely to recapitulate the naïve sequence whereas single-state design is more likely to recapitulate the mature sequence. The conclusion is that germline sequences possess high conformational flexibility.[49]

18	Adhikary et al.	2015	Biochemistry	Similar to #12 & #15, this paper uses ITC, 3PEPS, crystallography, and ELISA to analyze the thermodynamics and dynamics of **anti-MPTS antibodies**. The authors selectively isolate three antibodies with varying specificity for MPTS and other proteins. The authors find that the antibody with the few somatic mutations is the most polyspecific, but also the least dynamic. On the other hand, the antibody with the highest affinity for MPTS is the most dynamic. The authors conclude that affinity maturation can have divergent effects on dynamics towards antigen recognition.[8]
19	Li et al.	2015	PLoS Comp. Bio.	The authors assess the flexibility of three antibodies (**anti-fluorescein, anti-CD3, 48G7**) using MD to generate ensembles and a distance constraint model to evaluate flexibility. The authors note a significant amount of rigidity increases in the CDR H3 loop and flexibility increases in the CDR L2 loop. They believe these effects are compensatory.[50]
20	Davenport et al.	2016	Structure	This paper studies the effects of SHM on the dynamics of three **anti-HIV antibodies** using HXMS. The authors find that most stabilization occurred in the CDR L2, H2, and FW3. This contradicts previous studies. The authors rationalize the contradiction as arising due to the relative complexity of HIV antigen versus the previous studied antigen.[51]

Supplementary Table 5. Manually identified germlines.

PDB	Selected Germline
5ggs	IGKV3D-7*01
5ibu	IGHV5-51*01
5wuv	IGHV3-48*01
5w05	IGHV5-10-1*01
5w06	IGHV5-10-1*01
5uy3	IGHV1-8*01
5v7j	IGLV3-21*02
5b71	IGHV3-66*01

3 References

1. Tyka, M. D., Keedy, D. A., André, I., Dimaio, F., Song, Y., Richardson, D. C., Richardson, J. S. & Baker, D. Alternate states of proteins revealed by detailed energy landscape mapping. *J. Mol. Biol.* **405,** 607–18 (2011).

2. Nivon, L. G., Moretti, R. & Baker, D. A Pareto-Optimal Refinement Method for Protein Design Scaffolds. *PLoS One* **8,** e59004 (2013).

3. Conway, P., Tyka, M. D., DiMaio, F., Konerding, D. E. & Baker, D. Relaxation of backbone bond geometry improves protein energy landscape modeling. *Protein Sci.* **23,** 47–55 (2014).

4. Mandell, D. J., Coutsias, E. A. & Kortemme, T. Sub-angstrom accuracy in protein loop reconstruction by robotics-inspired conformational sampling. *Nat. Methods* **6,** 551–552 (2009).

5. Stein, A. & Kortemme, T. Improvements to Robotics-Inspired Conformational Sampling in Rosetta. *PLoS One* **8,** e63090 (2013).

6. Sivasubramanian, A., Sircar, A., Chaudhury, S. & Gray, J. J. Toward high-resolution homology modeling of antibody F v regions and application to antibody-antigen docking. *Proteins Struct. Funct. Bioinforma.* **74,** 497–514 (2009).

7. Weitzner, B. D., Jeliazkov, J. R., Lyskov, S., Marze, N., Kuroda, D., Frick, R., Adolf-Bryfogle, J., Biswas, N., Dunbrack Jr., R. L. & Gray, J. J. Modeling and docking of antibody structures with Rosetta. *Nat Protoc* **12,** 401–416 (2017).

8. Li, T., Tracka, M. B., Uddin, S., Casas-Finet, J., Jacobs, D. J. & Livesay, D. R. Rigidity Emerges during Antibody Evolution in Three Distinct Antibody Systems: Evidence from QSFR Analysis of Fab Fragments. *PLoS Comput Biol* **11,** e1004327 (2015).

9. Li, T., Verma, D., Tracka, M. B., Casas-Finet, J., Livesay, D. R. & Jacobs, D. J. Thermodynamic stability and flexibility characteristics of antibody fragment complexes. *Protein Pept Lett* **21,** 752–765 (2014).

10. Li, T., Tracka, M. B., Uddin, S., Casas-Finet, J., Jacobs, D. J. & Livesay, D. R. Redistribution of flexibility in stabilizing antibody fragment mutants follows Le Chatelier's principle. *PLoS One* **9,** e92870 (2014).

11. Srivastava, A., Tracka, M. B., Uddin, S., Casas-Finet, J., Livesay, D. R. & Jacobs, D. J. Mutations in Antibody Fragments Modulate Allosteric Response Via Hydrogen-Bond Network Fluctuations. *Biophys J* **110,** 1933–1942 (2016).

12. Yin, J., Mundorff, E. C., Yang, P. L., Wendt, K. U., Hanway, D., Stevens, R. C. & Schultz, P. G. A comparative analysis of the immunological evolution of antibody 28B4. *Biochemistry* **40,** 10764–10773 (2001).

13. Hsieh-Wilson, L. C., Schultz, P. G. & Stevenst, R. C. Insights into antibody catalysis: Structure of an oxygenation catalyst at 1.9-A resolution (crystal structure/catalytic antibody/oxidation). *Biochemistry* **93,** 5363–5367 (1996).

14. Yin, J., Mundorff, E. C., Yang, P. L., Wendt, | K Ulrich, Hanway, D., Stevens, R. C. & Schultz, P. G. A Comparative Analysis of the Immunological Evolution of Antibody 28B4. doi:10.1021/bi010536c

15. Ulrich, H. D., Mundorff, E., Santarsiero, B. D., Driggers, E. M., Stevens, R. C. & Sehulta, P. G. The interplay between binding energy and catalysis in the evolution of a catalytic antibody. *Nature* **389,** 271–275 (1997).

16. Mundorff, E. C., Hanson, M. A., Varvak, A., Ulrich, H., Schultz, P. G. & Stevens, R. C. Conformational effects in biological catalysis: An antibody-catalyzed oxy-Cope rearrangement. *Biochemistry* **39,** 627–632 (2000).

17. Nguyen, H. P., Seto, N. O. L., MacKenzie, C. R., Brade, L., Kosma, P., Brade, H. & Evans, S. V. Germline antibody recognition of distinct carbohydrate epitopes. *Nat. Struct. Biol.* **10,** 1019–1025 (2003).

18. Wedemayer, G. J., Patten, P. A., Wang, L. H., Schultz, P. G. & Stevens, R. C. Structural insights into the evolution of an antibody combining site. *Science* **276,** 1665–9 (1997).

19. Wedemayer, G. J., Wang, L. H., Patten, P. A., Schultz, P. G. & Stevens, R. C. Crystal structures of the free and liganded form of an esterolytic catalytic antibody. *J. Mol. Biol.* **268,** 390–400 (1997).

20. Patten, P. A., Gray, N. S., Yang, P. L., Marks, C. B., Wedemayer, G. J., Boniface, J. J., Stevens, R. C. & Schultz, P. G. The immunological evolution of catalysis. *Science (80-.).* **271,** 1086–1091 (1996).

21. Braden, B. C., Souchon, H., Eiselé, J. L., Bentley, G. A., Bhat, T. N., Navaza, J. & Poljak, R. J. Three-dimensional structures of the free and the antigen-complexed Fab from monoclonal anti-lysozyme antibody D44.1. *J. Mol. Biol.* **243,** 767–781 (1994).

22. Acierno, J. P., Braden, B. C., Klinke, S., Goldbaum, F. A. & Cauerhff, A. Affinity Maturation Increases the Stability and Plasticity of the Fv Domain of Anti-protein Antibodies. *J. Mol. Biol.* **374,** 130–146 (2007).

23. Cauerhff, A., Goldbaum, F. A. & Braden, B. C. Structural mechanism for affinity maturation of an anti-lysozyme antibody. *Proc. Natl. Acad. Sci.* **101,** 3539–3544 (2004).

24. Li, Y., Li, H., Yang, F., Smith-Gill, S. J. & Mariuzza, R. A. X-ray snapshots of the maturation of an antibody response to a protein antigen. *Nat. Struct. Mol. Biol.* **10,** 482–488 (2003).

25. Li, Y., Li, H., Smith-Gill, S. J. & Mariuzza, R. A. Three-dimensional structures of the free and antigen-bound Fab from monoclonal antilysozyme antibody HyHEL-63. *Biochemistry* **39,** 6296–6309 (2000).

26. Terzyan, S., Ramsland, P. A., Voss, E. W., Herron, J. N. & Edmundson, A. B. Three-dimensional structures of idiotypically related Fabs with intermediate and high affinity for fluorescein. *J. Mol. Biol.* **339,** 1141–1151 (2004).

27. Herron, J. N., He, X. -m, Mason, M. L., Voss, E. W. & Edmundson, A. B. Three-dimensional structure of a fluorescein–Fab complex crystallized in 2-methyl-2,4-pentanediol. *Proteins Struct. Funct. Bioinforma.* **5,** 271–280 (1989).

28. Schmidt, A. G., Xu, H., Khan, A. R., O'Donnell, T., Khurana, S., King, L. R., Manischewitz, J., Golding, H., Suphaphiphat, P., Carfi, A., Settembre, E. C., Dormitzer, P. R., Kepler, T. B., Zhang, R., Moody, M. A., Haynes, B. F., Liao, H.-X. X., Shaw, D. E. & Harrison, S. C. Preconfiguration of the antigen-binding site during affinity maturation of a broadly neutralizing influenza virus antibody. *Proc Natl Acad Sci U S A* **110,** 264–9 (2013).

29. Mouquet, H., Scharf, L., Euler, Z., Liu, Y., Eden, C., Scheid, J. F., Halper-Stromberg, A., Gnanapragasam, P. N. P., Spencer, D. I. R., Seaman, M. S., Schuitemaker, H., Feizi, T., Nussenzweig, M. C. & Bjorkman, P. J. Complex-type N-glycan recognition by potent broadly neutralizing HIV antibodies. *Proc. Natl. Acad. Sci.* **109,** E3268–E3277 (2012).

30. Scharf, L., West, A. P., Gao, H., Lee, T., Scheid, J. F., Nussenzweig, M. C., Bjorkman, P. J. & Diskin, R. Structural basis for HIV-1 gp120 recognition by a germ-line version of a broadly neutralizing antibody. *Proc. Natl. Acad. Sci.* **110,** 6049–6054 (2013).

31. Diskin, R., Scheid, J. F., Marcovecchio, P. M., West, A. P., Klein, F., Gao, H., Gnanapragasam, P. N. P., Abadir, A., Seaman, M. S., Nussenzweig, M. C. & Bjorkman, P. J. Increasing the Potency and Breadth of an HIV Antibody by Using Structure-Based Rational Design. *Science (80-.).* **334,** 1289–1293 (2011).

32. Jardine, J., Julien, J.-P., Menis, S., Ota, T., Kalyuzhniy, O., McGuire, A., Sok, D., Huang, P.-S., MacPherson, S., Jones, M., Nieusma, T., Mathison, J., Baker, D., Ward, A. B., Burton, D. R., Stamatatos, L., Nemazee, D., Wilson, I. A. & Schief, W. R. Rational HIV Immunogen Design to Target Specific Germline B Cell Receptors. *Science (80-.).* **340,** 711–716 (2013).

33. Wu, X., Zhang, Z., Schramm, C. A., Joyce, M. G., Do Kwon, Y., Zhou, T., Sheng, Z., Zhang, B., O'Dell, S., McKee, K., Georgiev, I. S., Chuang, G. Y., Longo, N. S., Lynch, R. M., Saunders, K. O., Soto, C., Srivatsan, S., Yang, Y., Bailer, R. T., Louder, M. K., Mullikin, J. C., Connors, M., Kwong, P. D., Mascola, J. R. & Shapiro, L. Maturation and diversity of the VRC01-antibody lineage over 15 years of chronic HIV-1 infection. *Cell* **161,** 480–485 (2015).

34. Wu, X., Zhou, T., Zhu, J., Zhang, B., Georgiev, I., Wang, C., Chen, X., Longo, N. S., Louder, M., McKee, K., O'Dell, S., Perfetto, S., Schmidt, S. D., Shi, W., Wu, L., Yang, Y., Yang, Z.-Y., Yang, Z., Zhang, Z., Bonsignori, M., Crump, J. A., Kapiga, S. H., Sam, N. E., Haynes, B. F., Simek, M., Burton, D. R., Koff, W. C., Doria-Rose, N. A., Connors, M., Mullikin, J. C., Nabel, G. J., Roederer, M., Shapiro, L., Kwong, P. D. & Mascola, J. R. Focused Evolution of HIV-1 Neutralizing Antibodies Revealed by Structures and Deep Sequencing. *Science (80-.).* **333,** 1593–1602 (2011).

35. Davenport, T. M., Gorman, J., Joyce, M. G., Zhou, T., Soto, C., Guttman, M., Moquin, S., Yang, Y., Zhang, B., Doria-Rose, N. A., Hu, S.-L., Mascola, J. R., Kwong, P. D. & Lee, K. K. Somatic Hypermutation-Induced Changes in the Structure and Dynamics of HIV-1 Broadly Neutralizing Antibodies. *Structure* **24,** 1346–1357 (2016).

36. Doria-Rose, N. A., Schramm, C. A., Gorman, J., Moore, P. L., Bhiman, J. N., DeKosky, B. J.,

Ernandes, M. J., Georgiev, I. S., Kim, H. J., Pancera, M., Staupe, R. P., Altae-Tran, H. R., Bailer, R. T., Crooks, E. T., Cupo, A., Druz, A., Garrett, N. J., Hoi, K. H., Kong, R., Louder, M. K., Longo, N. S., McKee, K., Nonyane, M., O'Dell, S., Roark, R. S., Rudicell, R. S., Schmidt, S. D., Sheward, D. J., Soto, C., Wibmer, C. K., Yang, Y., Zhang, Z., NISC Comparative Sequencing, N. C., Mullikin, J. C., Binley, J. M., Sanders, R. W., Wilson, I. A., Moore, J. P., Ward, A. B., Georgiou, G., Williamson, C., Abdool Karim, S. S., Morris, L., Kwong, P. D., Shapiro, L. & Mascola, J. R. Developmental pathway for potent V1V2-directed HIV-neutralizing antibodies. *Nature* **509,** 55–62 (2014).

37. Chong, L. T., Duan, Y., Wang, L., Massova, I. & Kollman, P. A. Molecular dynamics and free-energy calculations applied to affinity maturation in antibody 48G7. *Proc. Natl. Acad. Sci. U. S. A.* **96,** 14330–14335 (1999).

38. Manivel, V., Sahoo, N. C., Salunke, D. M. & Rao, K. V. . Maturation of an antibody response is governed by modulations in flexibility of the antigen-combining site. *Immunity* **13,** 611–620 (2000).

39. Jimenez, R., Salazar, G., Baldridge, K. K. & Romesberg, F. E. Flexibility and molecular recognition in the immune system. *Proc Natl Acad Sci U S A* **100,** 92–97 (2003).

40. Yin, J., Beuscher, A. E., Andryski, S. E., Stevens, R. C. & Schultz, P. G. Structural plasticity and the evolution of antibody affinity and specificity. *J. Mol. Biol.* **330,** 651–656 (2003).

41. Jimenez, R., Salazar, G., Yin, J., Joo, T. & Romesberg, F. E. Protein dynamics and the immunological evolution of molecular recognition. *Proc Natl Acad Sci U S A* **101,** 3803–3808 (2004).

42. Zimmermann, J. J., Oakman, E. L., Thorpe, I. F., Shi, X., Abbyad, P., Brooks, C. L., Boxer, S. G., Romesberg, F. E., Brooks 3rd, C. L., Boxer, S. G. & Romesberg, F. E. Antibody evolution constrains conformational heterogeneity by tailoring protein dynamics. *Proc Natl Acad Sci U S A* **103,** 13722–13727 (2006).

43. Thorpe, I. F., Brooks, C. L. & Brooks 3rd, C. L. Molecular evolution of affinity and flexibility in the immune system. *Proc. Natl. Acad. Sci. U. S. A.* **104,** 8821–8826 (2007).

44. Thielges, M. C., Zimmermann, J., Yu, W., Oda, M. & Romesberg, F. E. Exploring the energy landscape of antibody-antigen complexes: Protein dynamics, flexibility, and molecular recognition. *Biochemistry* **47,** 7237–7247 (2008).

45. Babor, M. & Kortemme, T. Multi-constraint computational design suggests that native sequences of germline antibody H3 loops are nearly optimal for conformational flexibility. *Proteins Struct. Funct. Bioinforma.* **75,** 846–858 (2009).

46. Wong, S. E., Sellers, B. D. & Jacobson, M. P. Effects of somatic mutations on CDR loop flexibility during affinity maturation. *Proteins Struct. Funct. Bioinforma.* **79,** 821–829 (2011).

47. Adhikary, R., Yu, W., Oda, M., Zimmermann, J. & Romesberg, F. E. Protein dynamics and the diversity of an antibody response. *J Biol Chem* **287,** 27139–27147 (2012).

48. Willis, J. R., Briney, B. S., DeLuca, S. L., Crowe, J. E., Meiler, J., Crowe Jr., J. E. & Meiler, J. Human germline antibody gene segments encode polyspecific antibodies. *PLoS Comput Biol* **9,** e1003045 (2013).

49. Adhikary, R., Yu, W., Oda, M., Walker, R. C., Chen, T., Stanfield, R. L., Wilson, I. A., Zimmermann, J. & Romesberg, F. E. Adaptive mutations alter antibody structure and dynamics during affinity maturation. *Biochemistry* **54,** 2085–2093 (2015).

50. Davenport, T. M., Gorman, J., Joyce, M. G., Zhou, T., Soto, C., Guttman, M., Moquin, S., Yang, Y., Zhang, B., Doria-Rose, N. A., Hu, S. L., Mascola, J. R., Kwong, P. D. & Lee, K. K. Somatic Hypermutation-Induced Changes in the Structure and Dynamics of HIV-1 Broadly Neutralizing Antibodies. *Structure* **24,** 1346–1357 (2016).

51. Yin, J., Andryski, S. E., Beuscher, A. E., Stevens, R. C. & Schultz, P. G. Structural evidence for substrate strain in antibody catalysis. *Proc. Natl. Acad. Sci. U. S. A.* **100,** 856–61 (2003).
